# Carcinogenesis and Reactive Oxygen Species Signaling: Interaction of the NADPH Oxidase NOX1–5 and Superoxide Dismutase 1–3 Signal Transduction Pathways

**DOI:** 10.1089/ars.2017.7268

**Published:** 2019-02-11

**Authors:** Alessia Parascandolo, Mikko O. Laukkanen

**Affiliations:** IRCCS SDN, Naples, Italy.

**Keywords:** redox signaling, NADPH oxidase NOX, superoxide dismutase, tyrosine kinase receptor, G protein-coupled receptor

## Abstract

***Significance:*** Reduction/oxidation (redox) balance could be defined as an even distribution of reduction and oxidation complementary processes and their reaction end products. There is a consensus that aberrant levels of reactive oxygen species (ROS), commonly observed in cancer, stimulate primary cell immortalization and progression of carcinogenesis. However, the mechanism how different ROS regulate redox balance is not completely understood.

***Recent Advances:*** In the current review, we have summarized the main signaling cascades inducing NADPH oxidase NOX1–5 and superoxide dismutase (SOD) 1–3 expression and their connection to cell proliferation, immortalization, transformation, and CD34^+^ cell differentiation in thyroid, colon, lung, breast, and hematological cancers.

***Critical Issues:*** Interestingly, many of the signaling pathways activating redox enzymes or mediating the effect of ROS are common, such as pathways initiated from G protein-coupled receptors and tyrosine kinase receptors involving protein kinase A, phospholipase C, calcium, and small GTPase signaling molecules.

***Future Directions:*** The clarification of interaction of signal transduction pathways could explain how cells regulate redox balance and may even provide means to inhibit the accumulation of harmful levels of ROS in human pathologies.

**Table:** 

**Table of Contents**	
I. Introduction	444
A. Superoxide anion and hydrogen peroxide	444
B. Concentration-dependent effect of H_2_O_2_ and O_2_^•−^ on signal transduction	445
II. NADPH Oxidase NOX1–5 Family	446
III. NADPH Oxidase NOX1	446
A. NADPH oxidase NOX1 in tumorigenesis	446
IV. NADPH Oxidase NOX2	448
A. NADPH oxidase NOX2 complex formation	448
V. NADPH Oxidase NOX3	449
VI. NADPH Oxidase NOX4	449
A. NADPH oxidase NOX4 in tumorigenesis	449
VII. NADPH Oxidase NOX5	450
A. NADPH oxidase NOX5 in tumorigenesis	451
VIII. SOD1–3 Family	451
IX. Copper Zinc SOD, CuZnSOD, SOD1	451
A. SOD1 in tumorigenesis	452
X. Manganese SOD, MnSOD, SOD2	453
A. SOD2 in tumorigenesis	453
XI. Extracellular SOD, EC-SOD, SOD3	454
A. SOD3 in tumorigenesis	455
XII. Interaction of NOX1–5- and SOD1–3-Associated Signaling	456
A. GPCR signaling	456
B. Ca^2+^ signaling	458
C. RTK and small GTPase signaling	458
D. Oncogene signaling	459
XIII. Altered ROS Levels Are Associated with Progression of Tumorigenesis	459
XIV. ROS in Thyroid Cancer	460
A. Redox-related signaling in thyroid tumorigenesis	460
B. *NOX1–5* and *SOD1–3* expression in thyroid cancer	461
C. Percentage change in redox gene expression in PTC	463
XV. ROS in Colon Cancer	464
A. Progression of colon cancer	464
B. WNT signaling in the normal colon and in colon cancer development	465
C. *NOX1–5* and *SOD1–3* gene expression in colon tumorigenesis	465
XVI. ROS in Breast Cancer	466
A. ROS-related characteristics of breast cancer	466
B. *NOX1–5* and *SOD1–3* gene expression in breast tumorigenesis	467
XVII. ROS in Lung Cancer	467
A. ROS-related characteristics of lung cancer	467
B. *NOX1–5* and *SOD1–3* gene expression in lung tumorigenesis	469
XVIII. ROS in Hematological Cancers	469
A. ROS in CD34 HSC differentiation	469
B. ROS in hematological cancers and therapy	470
XIX. Summary and Conclusions	471

## I. Introduction

### A. Superoxide anion and hydrogen peroxide

Reactive oxygen species (ROS), a heterogeneous group of reactive oxygen derivatives, are involved in cellular signal transduction events regulating growth, differentiation, survival, and apoptosis. The effect of ROS on oxidative cell signaling depends on the type of ROS produced, concentration of ROS, localization of ROS, and persistence of ROS production. Increased or decreased production of ROS has a drastic impact on cell fate, thus reflecting the importance of ROS balance for cellular signal transduction.

Superoxide anion (O_2_^•−^), produced by NADPH oxidases, and hydrogen peroxide (H_2_O_2_), produced by superoxide dismutases (SODs) and by NADPH oxidases, represent intensively investigated ROS. Both ROS function as second messengers in cellular signaling, being able to activate or inactivate signaling pathways, thus regulating the phosphorylation of tyrosine kinase receptors (RTKs) and downstream signaling molecules. ROS affect virtually all normal and pathological conditions, including the function of the normal and injury-related cardiovascular systems (307, 391), hematopoiesis (44, 208), cancer (90), fibrotic diseases (40, 382), aging (90, 98), neurodegeneration (8), cellular senescence (98), apoptosis, and cell death (254, 299).

**FIG. 1.**
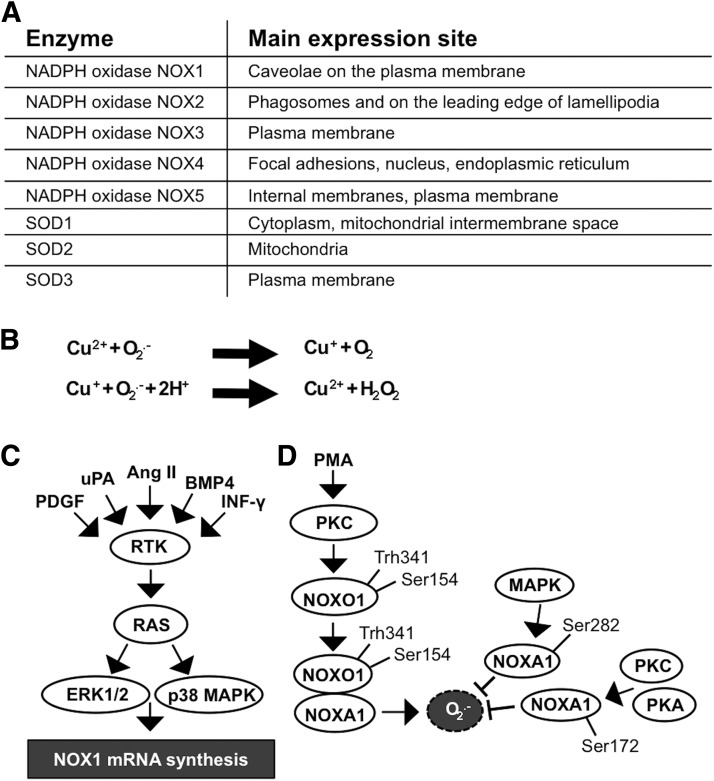
**Redox enzyme NADPH oxidase 1–5 and SOD1–3 expression is influenced by various factors in different cellular localizations. (A)** Primary expression sites at cell membranes and cellular organelles. **(B)** O_2_^•−^ is dismutated to H_2_O_2_ in two half-reactions. **(C)** Stimulation of NOX1 expression. RTK activation induces RAS-ERK1/2 and RAS-p38MAPK signaling pathways, thereby stimulating *NOX1* mRNA synthesis. **(D)** Mitogen stimulation of the PKC pathway induces NOXO1 phosphorylation at Thr154 and Thr341 causing dimer formation with NOXA1 and consequent O_2_^•−^ formation, which is attenuated by MAPK, PKC, and PKA-induced phosphorylation of NOXA1 at Ser172 and Ser282. H_2_O_2_, hydrogen peroxide; mRNA, messenger RNA; NOXA1, NADPH oxidase activator 1 subunit; NOXO1, NADPH oxidase organizer 1 subunit; O_2_^•−^, superoxide anion; PKA/AKT, protein kinase A; PKC, protein kinase C; redox, reduction/oxidation; RTK, tyrosine kinase receptor; SOD, superoxide dismutase.

The location of NADPH oxidases and SOD enzymes in different cellular membranes and organelles (31, 163, 314) may influence the physiological roles of these molecules in cells and the signaling pathways regulating cellular functions (Fig. 1A).

O_2_^•−^ is a short-lived, highly reactive radical that, in aberrant levels, causes a high number of modifications in cellular functions. Although the NADPH oxidase family of NOX enzymes is an intensively studied source of O_2_^•−^ ROS, ROS are also produced from other cellular organelles, such as those of the mitochondrial respiratory chain, composed of complexes I–IV. In mitochondria, the O_2_^•−^ radical is produced by complex I, the largest unit in the mitochondrial respiratory chain, which oxidizes NADH to NAD to produce ubiquinone and simultaneously release protons that contribute to ATP production (325, 381).

During electron transport, complex III produces four protons that are released into the intermembrane space, creating a transmembrane proton gradient that is later used by ATP synthase to synthesize ATP, and reduces cytochrome C levels, releasing electrons to complex IV. In addition, there is a premature leakage of a small portion of electrons from complex III that, in certain cases, may react with oxygen, resulting in O_2_^•−^ formation (6, 68, 160).

The catalysis of O_2_^•−^ to H_2_O_2_ can be spontaneous or catalyzed by SOD enzymes in two half reactions (Fig. 1B). The half-life of H_2_O_2_ is markedly longer than that of O_2_^•−^. Although in the tissue environment the half-life of H_2_O_2_ is only 1–3 ms (249), in the cell culture environment it may take even 40 min for a single 20-μ*M* dose of H_2_O_2_ to be completely erased (366). When studying the impact of H_2_O_2_ on cellular homeostasis *in vitro*, it is important to note that the extracellular H_2_O_2_ concentration is ∼10-fold higher than the intracellular concentration (336), indicating a regulatory mechanism that controls H_2_O_2_ transport into the cells. In general, in all aerobic organisms, the physiological intracellular H_2_O_2_ concentration varies from 0.001 to 0.5 μ*M* up to a maximum of 0.7 μ*M* (340). Although H_2_O_2_ may passively diffuse through membrane structures, a recent article demonstrated that H_2_O_2_ passes the cell membrane, mainly through the aquaporin 3 (AQP3) integral membrane protein channel (239). The observation suggests that AQP3 functions as a regulator of the H_2_O_2_ concentration close to cell membrane-associated signaling molecules, such as RTKs and cell membrane-associated nonreceptor kinases.

### B. Concentration-dependent effect of H_2_O_2_ and O_2_^•−^ on signal transduction

In mammalian cells, such as thyrocytes and hepatocytes, and even in some microbes, H_2_O_2_ has been shown to have growth and survival supportive characteristics at low physiological intracellular levels (0.001–0.7 μ*M*), whereas higher levels (20–200 μ*M*) induce growth arrest, and levels above that eventually cause cell death (118, 336, 339), thus suggesting a dose-dependent response in signal transduction, cell growth, and survival. At high concentrations, H_2_O_2_, similar to other ROS, reacts with various cellular macromolecules, causing oxidative stress, DNA damage, mutagenesis, and apoptosis; however, at lower physiological concentrations, H_2_O_2_ modifies cellular signal transduction by stimulating or inhibiting signaling molecule activation (339, 340).

The mechanism underlying how ROS activate cell surface RTKs and downstream signal transduction is based on the coordinated action of RTKs and protein tyrosine phosphatases (PTPs). RTK activation is controlled by PTPs, which inactivate phosphorylated tyrosine amino acids in RTKs simply by removing the phosphate groups. H_2_O_2_-derived activation of RTKs is based on its ability to induce the oxidative inactivation of PTPs by modifying the catalytic site cysteine amino acids. Cysteine residues are reversibly oxidized to sulfenic acid (S-OH), followed by the removal of oxygen in a reaction resulting in cyclic sulfenamide formation. Sulfenamide formation causes a structural change in the catalytic site, exposing oxidatively inactivated cysteine groups to the solvent position. In the newly formed position, the cysteine residues are available as reducing agents, returning PTPs back to the active form (267, 354).

It is noteworthy that the response of H_2_O_2_ to cellular functions is not only location and concentration dependent but can also be based on the duration of oxidative stress (231, 250, 372). Treatment of primary fibroblasts by adding 10 μ*M* H_2_O_2_ to the cell culture medium every 3 days for 2 weeks to model low-level persistent cell stress reduced the DNA damage repair capacity, causing the accumulation of DNA damage and resulting in telomere shortening, eventually inducing irreversible growth arrest and senescence (82). Therefore, a low level of oxidative stress, which initially may induce cell survival mechanisms, in the long-term may cause irreversible changes in signal transduction, eventually destroying the cell or, alternatively, initiating the cell transformation process.

In general, the imbalanced production of ROS, especially H_2_O_2_, may affect signal transduction by promoting cancer cell survival, proliferation, migration, and drug resistance (21). Although cancer cells become adapted to increased ROS levels, the aberrant supraphysiological levels of either O_2_^•−^ or H_2_O_2_ markedly influence cell survival by reducing cell cycling and cell proliferation. Therefore, increased ROS levels are frequently used as cytotoxins in cancer patients (13, 23).

## II. NADPH Oxidase NOX1–5 Family

Characteristically, NADPH oxidase isoforms are heteromeric multiunit structures that are activated by the sequential assembly of subunits at cell membranes (31, 213). The most extensively studied isoform contains the phagocytic NADPH oxidase gp91^phox^ (phox, phagocytic oxidase) subunit, known as NOX2, which is expressed in monocytes/macrophages and granulocytes. The phagocytic NADPH oxidase NOX2-dependent respiratory/oxidative burst is one of the main sources of O_2_^•−^, reaching even 25 μ*M* inside a phagolysosome, which is formed from the cell membrane-composed phagosome (133, 326). Phagosome formation occurs in ∼20 s (318), after which the microbes containing the phagosome are internalized in the cell, fused with lysosomes to form phagolysosomes where the microbes are destroyed by oxidative burst-derived ROS and the action of antimicrobial factors (333).

Cellular responses to stimuli may activate oxidative burst through the calcium-protein kinase C (Ca^2+^-PKC) signaling pathway, leading to increased ROS production by NOX2 assembly (described below) at the phagolysosome membrane [reviewed in Ginis and Tauber (108) and Slauch (333)]. Although the NADPH oxidase NOX2 is commonly described in phagocytic cells, it is also expressed in nonphagocytic cells, such as cardiomyocytes (181) and leukemia cells (220). The other nonphagocytic enzymes NADPH oxidase NOX1, NADPH oxidase NOX3, NADPH oxidase NOX4, and NADPH oxidase NOX5, which produce less O_2_^•−^ than phagocytic NADPH oxidase NOX2, use a similar principle in O_2_^•−^ production, although the multiunit structure of the enzyme varies [reviewed in Bedard and Krause (19) and Panday *et al.* (270)].

Nonphagocytic NADPH oxidase NOX1-, NADPH oxidase NOX4-, and NADPH oxidase NOX5-derived ROS play a prominent role in the initiation and progression of cancer through the regulation of cellular signal transduction pathways (114, 159, 328, 342) and through modulation of the growth modulation signaling that supports the autonomous growth of cells, angiogenesis, invasion, and metastasis (240, 260, 352).

## III. NADPH Oxidase NOX1

The NADPH oxidase NOX1 complex is composed of the NOX1 subunit (gp91^phox^ homologue) (342), NADPH oxidase organizer 1 subunit (NOXO1), which is a homologue of p47^phox^, NADPH oxidase activator 1 subunit (NOXA1), which is a homologue of p67^phox^ (102), p22^phox^ subunit (7), and small GTPase RAC1 subunit (242). NOXO1 colocalizes with NOX1 to resting cell plasma membrane by binding to the phosphatidylinositol (PtdIns) lipids PtdIns 3,5-P2, PtdIns 5-P, and PtdIns 4-P with the Phox homology (PX) domain, hence suggesting both activation and localization roles for NOXO1 (51). NOX1 expression is most prominent in the colon where it is induced by factors representing various cellular effectors that increase both messenger RNA (mRNA) and protein expression, thereby stimulating O_2_^•−^ production. The NOX1 inducers include various RTK ligands, such platelet-derived growth factor (PDGF) (188), serine protease urokinase plasminogen activator (uPA) (237), peptide hormone angiotensin II (Ang II) (188), and cytokines—for example, bone morphogenic protein 4 (BMP4) (337) and interferon gamma (IFN-γ) (88, 183) (Fig. 1C).

The assembly and activation of the NADPH oxidase complex NOX1 are initiated by NOXO1 phosphorylation at Ser154 by cAMP-stimulated protein kinase A (PKA) (72, 388). Phorbol 12-myristate 13-acetate (PMA), a potent mitogen, has been demonstrated to activate PKC, which in turn may induce the phosphorylation of NOXO1 at Thr341/Ser154, resulting in the interaction of NOXO1 with NOXA1 and the simultaneous increase of O_2_^•−^ production (388). The proline-rich region at the N-terminal end of NOXA1 can bind to SRC downstream targets tyrosine kinase substrate with 4 SRC homology 3 (SH3) domains (TKS4) and tyrosine kinase substrate with 5 SH3 domains (TKS5), which enhances NOXA1 binding to NOX1 and causes consequent localization to invapodia and increased ROS production (106).

The extracellular stimulus-induced mitogenic RAS-MEK-ERK and PKC-PKA pathways control the hyperactivation of radical production through increased NOX1 gene expression and phosphorylation of NOXA1 at Ser282 and Ser172, thereby decreasing the affinity of RAC1 for NOX1 (5, 182) (Fig. 1D).

### A. NADPH oxidase NOX1 in tumorigenesis

**FIG. 2.**
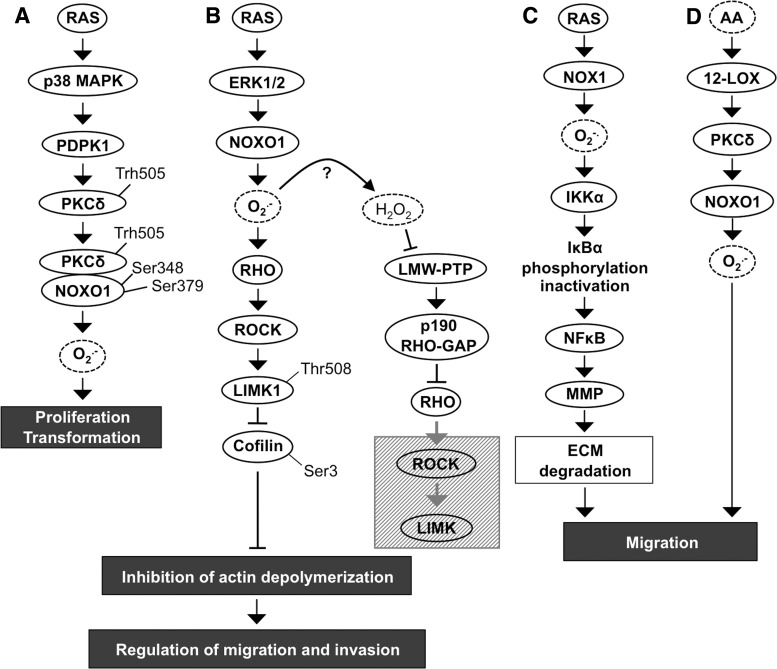
**RAS induces the proliferation and migration of cancer cells** ***via*** **NOX1. (A)** The RAS-p38MAPK signaling pathway induces PKCδ phosphorylation at Thr505, which causes consequent PKCδ-NOXO1 dimerization and phosphorylation of NOXO1 at Ser348 and Ser379. NADPH oxidase NOX1 produces O_2_^•−^ thereby stimulating cancer cell migration. **(B)** RAS-ERK1/2 induced NOXO1 activation and increased O_2_^•−^-stimulated signaling downstream to the RHO-ROCK-LIMK1 pathway that then inhibits cofilin by phosphorylation at Ser3 and consequently impacts actin depolymerization. H_2_O_2_ produced after activation of NOXO1 may inactivate phosphotyrosine phosphatase LMW-PTP. Consequent increased expression of p190 RHO GAP enhances GTP removal from RHO small GTPase downregulating the downstream ROCK-LIMK1 pathway. **(C)** RAS-induced signaling through IKKα-NFκB induces local cancer cell migration by MMP activation and ECM degradation. **(D)** Migration is also stimulated by arachidonic acid and 12-lipoxygenase-induced PKCδ signaling that activates NOXO1 and increases O_2_^•−^ production. ECM, extracellular matrix; GAP, small GTPase activator protein; IKKα, inhibitor of nuclear factor kappa-B kinase subunit α; LIMK, LIM kinase; LMW-PTP, low-molecular-weight phosphotyrosine phosphatase; MMP, matrix metalloproteinase; NFκB, nuclear factor kappa-light-chain-enhancer of activated B cells; PTP, protein tyrosine phosphatase; ROCK, RHO-associated, coiled-coil-containing protein kinase.

The role of the NADPH oxidase NOX1 in cell transformation is indisputable. Overexpression of this enzyme has been shown to promote the carcinogenic conversion of NIH-3T3 fibroblasts, causing morphological changes, increased anchorage-independent growth, and increased tumor formation *in vivo* (240, 342). However, it has been suggested that NADPH oxidase NOX1 plays a crucial role only in the initiation of tumorigenesis, as its expression is limited to the early stages of carcinogenesis and is downregulated in advanced cancers, excluding colon cancer (101, 240). NADPH oxidase NOX1-induced tumorigenesis is mediated primarily by a signal transduction pathway composed of mutated K-RASV12-stimulated p38MAPK, 3-phosphoinositide-dependent protein kinase-1 (PDPK1), and PKC delta (PKCδ) (Fig. 2A).

Phosphorylated p38MAPK activates PDPK1, a serine/threonine kinase that functions upstream of phosphatidylinositol-4,5-bisphosphate 3-kinase-protein kinase A (PI3K-PKA/AKT) pathway, p70S6 kinase, p90S6 kinase, p21-activated kinase (PAK1), and PKC. Activated PDPK1 phosphorylates PKCδ at Thr505 allowing the direct interaction of PKCδ with the SH region of the p47^phox^ homologue NOXO1, which is consequently phosphorylated at Ser348 and Ser379. Activation of the p47^phox^ homologue NOXO1 induces NADPH oxidase NOX1 complex assembly and the initiation of ROS production, resulting in increased anchorage-independent growth *in vitro* and increased tumorigenesis *in vivo* (196, 278) (Fig. 2A).

Although the function of p38MAPK in tumorigenesis is still under debate, the increased p38MAPK phosphorylation in breast, lung, and thyroid cancer patients correlates with lymph node metastasis, tamoxifen resistance, and poor prognosis (367). Paradoxically, several studies have reported tumor suppressor properties of p38MAPK, which correlate to the ROS concentration in the cells (76, 214, 367), therefore suggesting that the role of p38MAPK in tumorigenesis may depend on upstream/downstream mediators and their function.

In addition to the transformation effect in colon, breast, prostate, lung, and in ovarian cancer cells (5, 102, 240, 342), the increased tumorigenicity of NADPH oxidase NOX1 is mediated through angiogenic stimuli by the upregulation of vascular endothelial growth factor (VEGF) expression and the phosphorylation of VEGF receptors VEGFR-1 and VEGFR-2 (12). VEGF, an efficient promoter of endothelial cell migration in angiogenesis, has been demonstrated to stimulate breast carcinoma invasion utilizing chemokine receptor C-X-C chemokine receptor type 4 (CXCR4) affinity toward stromal-derived factor 1 (SDF-1) (14). Notably, increased ROS production by RAS-MEK1/2-ERK1/2-NADPH oxidase NOX1 upregulates small GTPase RHO and its direct downstream target RHO-associated, coiled-coil-containing protein kinase (ROCK) (Fig. 2B). Several reports have demonstrated ROCK-driven phosphorylation of LIM kinase (LIMK) that then through downstream cofilin, an actin binding protein, regulates disassembly of actin filaments. Phosphorylation of LIMK1 at Thr508 causes inactivation of cofilin by Ser3 phosphorylation, inhibition of actin depolymerization, and accumulation of actin fibers. Nonphosphorylated cofilin resides in the cellular protrusions of migrating cancer cells, whereas Ser3-phosphorylated inactive cofilin is distributed throughout the cytoplasm, thereby affecting cellular motility. Interestingly, H_2_O_2_ can inactivate low-molecular-weight phosphotyrosine phosphatase (LMW-PTP), which results in increased p190RHO-small GTPase activator protein (GAP) production and subsequent inactivation of RHO-ROCK-LIMK pathway (30, 212, 268, 281, 300, 330) (Fig. 2B).

RAS regulates cancer cell migration also through the inhibitor of nuclear factor kappa-B kinase subunit α (IKKα)-nuclear factor kappa-light-chain-enhancer of activated B cell (NFκB) pathway. NADPH oxidase NOX1 causes increased activity of IKKα, which phosphorylates IκBα (nuclear factor of kappa light polypeptide gene enhancer in B cell inhibitor α), an inhibitor of NFκB. Phosphorylation induces the degradation of IκBα, increasing NFκB activity, which in turn augments matrix metalloproteinase 9 (MMP9) expression and initiates extracellular matrix (ECM) degradation with consequent cancer cell invasion (330). Therefore, NOX1-driven invasion of cancer cells is based on the disruption of focal adhesions and increased expression of ECM-degrading enzymes, allowing cells to migrate locally and invade through ECM to initiate metastasis (Fig. 2C). In addition, the arachidonic acid (AA)-induced AA-12-lipoxygenase (LOX)-PKC-NOX1 pathway has been shown to decrease α_2_β_1_ integrin expression in cell membranes, eventually causing the loss of actin stress fibers and α_2_β_1_ integrins in cell membranes and therefore abrogating focal adhesions. The NADPH oxidase NOX1-stimulated local cancer cell migration along collagen I fibers depends on the NADPH oxidase NOX1-induced oxidative burst caused by AA-activated 12-LOX, which results in phosphorylation of PKC. Activated PKC phosphorylates the NOXO1 subunit, stimulating NOX1 assembly and thus increasing ROS production (308, 330) (Fig. 2D).

## IV. NADPH Oxidase NOX2

### A. NADPH oxidase NOX2 complex formation

NADPH oxidase NOX2, commonly referred by the catalytic subunit NOX2, is the most thoroughly characterized member of the NADPH oxidase family. Although NOX2 is mainly expressed in inflammatory cells involved in various biological functions, for example, in the host defense against invading microbes, it is frequently observed in tissues, such as cardiac muscle (168, 181). Signal transduction studies have shown that NADPH oxidase NOX2 complex formation commits small GTPase proteins and G protein-coupled receptor (GPCR), key signaling molecules mediating the extracellular stimuli into the intracellular signal transduction network, as a subunit of NADPH oxidase and as an activator of NADPH oxidase complex formation, respectively.

**FIG. 3.**
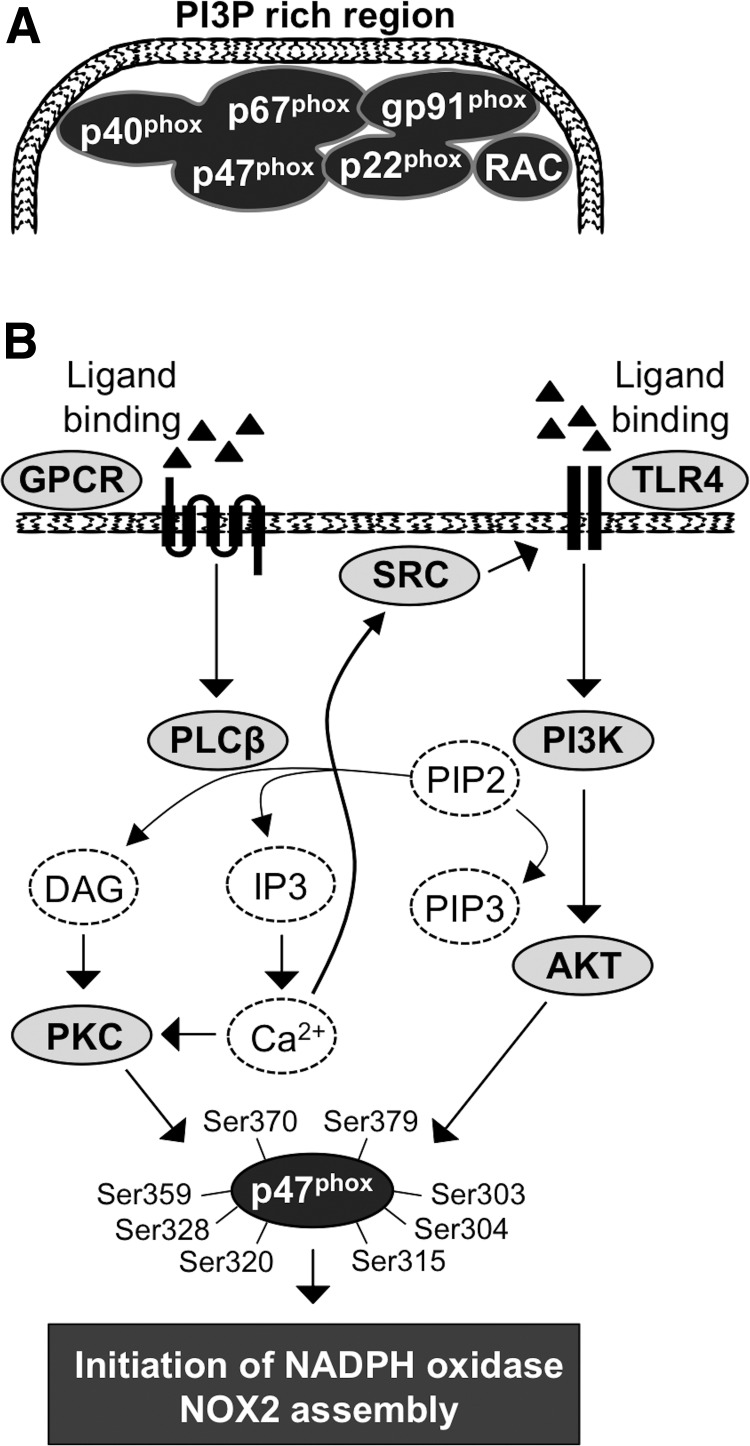
**Activation of NADPH oxidase NOX2 assembly. (A)** NADPH oxidase NOX2 is composed of a p40^phox^-p67^phox^-p47^phox^ heterotrimer and a p22^phox^-gp91^phox^ dimer that is activated at the cell membrane by association of small GTPase RAC into the complex. **(B)** GPCR activation stimulates PLCβ-DAG/IP3-Ca^2+^-PKC signaling that phosphorylates p47^phox^. Ca^2+^ may alternatively phosphorylate the SRC oncogene followed by activation of the TLR4-PI3K-AKT pathway, which further stimulates p47^phox^ subunit phosphorylation. Ca^2+^, calcium; Ca^2+^-PKC, calcium-protein kinase C; DAG, diacylglycerol; GPCR, G protein-coupled receptor; IP3, inositol triphosphate; PI3K, phosphatidylinositol-4,5-bisphosphate 3-kinase; PLC, phospholipase C; TLR4, Toll-like receptor 4.

In the resting-state cell, NADPH oxidase NOX2 units are organized into three different groups: (i) NOX2/gp91^phox^ and p22^phox^, which form a membrane-bound flavocytochrome b588 (cyt b588) (280); (ii) p47^phox^, p67^phox^, and p40^phox^, which are located in the cytosol as a heterotrimer attached to each other at their C-terminal ends (95, 203); (iii) the third component is small GTPase RAC1 (expressed in macrophages) (287) or RAC2 (expressed in granulocytes) (86, 130) (Fig. 3A). Phosphorylation of p47^phox^ initiates conformational changes in the p47^phox^ structure and the assembly of the NADPH oxidase NOX2 multistructure by transporting the p47^phox^-p67^phox^-p40^phox^ heterotrimer to the cell membrane (85, 140).

At the cell membrane, the heterotrimer is organized to enable the assembly of activated NADPH oxidase and the oxidative burst in the following steps: (i) the p47^phox^ SH3 domain interacts with the cyt b588 subunit p22^phox^ C-terminal proline-rich region (343); (ii) p67^phox^ binds to the cyt b588 gp91^phox^ subunit NADPH binding domain and functions as a regulator of oxidase activity (211); (iii) p40^phox^ binds to PI3P at PI3P-rich membrane regions, thus strengthening the interaction of the membrane and NAPDH oxidase complex in the presence of phosphorylated PI3K (359, 360) (Fig. 3A); and (iv) the final activation of the NADPH oxidase NOX2 complex occurs by the interaction of activated GTP-bound RAC with the p67^phox^ N-terminal region, enhancing gp91 subunit O_2_^•−^ production (176). RAC is an important regulatory factor in electron transfer from NADPH to flavin adenine dinucleotide (FAD) and consequently from FAD to heme groups in the gp91^phox^ catalytic center (75). More specifically, the association of small GTPase RAC activates the two-step electron transfer reaction: (i) electron transfer from NADPH to FAD, which results in the formation of NADP^+^ and FADH_2_ and (ii) electron transfer to cyt b588-associated heme and molecular oxygen, resulting in the formation of H^+^ and O_2_^•−^ (75).

In response to stimuli, the activated phagocytic NOX2 (gp91^phox^ unit of the complex), which contains one FAD and two nonidentical heme molecules, gradually catalyzes O_2_ to O_2_^•−^. The oxidative burst is initiated by electron transfer from NADPH to FAD, followed by electron transfer to the heme group, which, in turn, reduces O_2_ to O_2_^•−^ (19, 64). Activation of the oxidative burst is a multistep process stimulated by various signals, such as bacterial lipopolysaccharide (LPS), inflammatory cytokines, and platelet-activating factor (PAF), which can stimulate the activation of GPCR and Toll-like receptor 4 (TLR4) (134, 371). Ligand binding induces a conformational change in GPCR, eventually promoting the GDP switch to GTP, causing the activation of G proteins associated with GPCR and initiating the downstream signaling cascade (349).

TLR4 belongs to the type-1 transmembrane receptor family that is important in activation of NADPH NOX2 complex assembly (45) (Fig. 3B). In addition, TLR4 stimulates NFκB signal transduction pathway that activates tumor promoting molecules, such as VEGF-A, cyclooxygenase 2 (COX2), interleukins IL-6 and IL-8, and MMP9, which all increase cancer cell survival, immune escape, and increased metastasis (295).

Activation of GPCR stimulates the Gq heterotrimeric G protein subunit, which initiates phospholipase C β (PLCβ)-driven hydrolysis of membrane-bound phosphatidylinositol (4,5)-biphosphate (PIP2), resulting in the synthesis of diacylglycerol (DAG) and inositol triphosphate (IP3). DAG recruits PKC to the cell membrane, and IP3 promotes calcium (Ca^2+^) channel opening with a consequent temporal intracellular increase of Ca^2+^, which further enhances the activation of PKC, a crucially important kinase in the initiation of NADPH oxidase complex assembly (24, 277) (Fig. 3B). Several studies have demonstrated that increased intracellular Ca^2+^ uptake activates the phosphorylation of the proto-oncogene SRC.

The SRC family of protein tyrosine kinases, which comprises nine members (SRC, YES, LYN, YRK, BLK, FYN HCK, FGR, and LCK), interacts with RTKs and GPCRs, mediating their signaling to downstream networks. As mentioned above, the activation of SRC increases the phosphorylation of TLR4, which enhances PI3K-AKT pathway signal transduction, causing the phosphorylation of the NADPH oxidase regulatory subunit p47^phox^ together with PKC at serines 303, 304, 315, 320, 328, 359, 370, and 379 (45, 57, 92, 140, 253, 270, 334). More specifically, PI3K class I that is activated by downstream signaling from GPCRs and RTKs uses PIP2 as a precursor for the production of phosphatidylinositol (3,4,5)-triphosphate (PIP3), which then activates AKT immediately downstream of PI3K.

Interestingly, PI3K has been suggested to be activated simultaneously with PLC, resulting in simultaneous catalysis of PIP2 and consequent synthesis of PIP3, DAG, and IP3 (256, 294, 316) (Fig. 3B).

## V. NADPH Oxidase NOX3

Similar to NOX1 and NOX2, NOX3 contains the catalytic subunit gp91^phox^. The oxidative burst by NADPH oxidase NOX3 is stimulated by regulatory subunits p47*phox* and p67*phox*, or by subunits NOXO1 and NOXA1, thus showing compatibility for both NOXO and phox proteins. In addition, p22 *phox* is required for O_2_^•−^ production (52, 358). NOX3 is mostly expressed in the inner ear, fetal kidney, liver, lung, and spleen but is detected also at low mRNA levels in adult colon tissue (50, 269), suggesting a role for NOX3 in ontogenesis, findings that were corroborated by a study showing a crucial role of NADPH oxidase NOX3 in otoconial morphogenesis in the inner ear. The authors hypothesized that otoconin bound to membrane structure phospholipids undergoes NADPH oxidase NOX3-catalyzed conformational change. In the absence of NADPH oxidase NOX3, otoconin morphogenesis is defective. Abnormalities in otoconia may cause symptoms similar to those of vertigo (269).

Although the functional role of NADPH oxidase NOX3 is reported only in otoconia morphogenesis, there may be an association with the primitive cellular phenotype in general because *NOX3* mRNA expression is moderately augmented in undifferentiated ovarian teratocarcinoma and adenocarcinoma cancer cells (50).

## VI. NADPH Oxidase NOX4

NOX4 expression and function are linked to primary cell transformation, fibrosis, and cardiovascular diseases (25, 47, 65, 156, 389). The NOX4 isoform of NADPH oxidase comprises p22^phox^, polymerase delta-interacting protein 2 (209), and TKS5 (74). According to a recent work, small GTPase RAC1 can associate with the NADPH oxidase NOX4 complex, but it is not needed for full NOX4 activation (236). The most striking difference between NOX4 and other isoforms is the high concentration of H_2_O_2_ produced by the enzyme.

Although H_2_O_2_ synthesis by NADPH oxidase NOX4 has not been completely characterized, it has been suggested that the outer membrane E-loop may produce protons (H^+^), which then permit increased spontaneous dismutation of O_2_^•−^ to H_2_O_2_ (345). The same kinds of E-loop structures, although shorter, exist also in NOX1 and NOX2 NADPH oxidases, which, however, do not possess similar dismutase activity (345). Therefore, further studies are required to dissect whether O_2_^•−^ catalysis to H_2_O_2_ is purely spontaneous, based on the NADPH oxidase NOX4-associated subunit or enzyme.

### A. NADPH oxidase NOX4 in tumorigenesis

Increased NOX4 expression has been frequently connected to cell transformation. Recent studies suggested that in acute myeloid leukemia (AML) cells, mutated FMS-like tyrosine kinase 3 with internal tandem duplications (FLT3-ITDs) increases the synthesis of transcription factor signal transducer and activator of transcription 5 (STAT5), which binds to the *NOX4* promoter region at IFN-γ activated sequence elements, activating *NOX4* gene transcription. The FLT3-ITD oncogene also induces the phosphorylation of PI3K-AKT that then activates the p22^phox^ subunit with consequent NADPH oxidase NOX4 assembly.

NADPH oxidase NOX4-derived O_2_^•−^ and H_2_O_2_ then contribute to the inactivation of protein tyrosine phosphatase, receptor type J (PTPRJ, also known as DEP-1), a transmembrane PTP that negatively regulates FLT3 signaling activity, and the transformation of primary cells (155, 156). Hence, NADPH oxidase NOX4-derived inactivation of PTPRJ could explain increased FLT3 activation and the consequent transformation of hematopoietic cells. Furthermore, FLT3-ITD overexpression has been shown to increase DNA damage, such as DNA oxidation modification and double-strand breaks; this damage contributes to the initiation and progression of carcinogenesis, resulting in an aggressive cancer cell phenotype and enhanced drug resistance with consequent disease relapse. Mechanistically, p22^phox^ has been suggested to mediate FLT3-ITD-stimulated ROS production by activating NOX4 in the nuclear membrane of leukemia cells, thereby causing increased nuclear ROS contents and consequent DNA damage (155, 156, 338). Additional evidence for the tumor-promoting role of NADPH oxidase NOX4 was offered by a study demonstrating NADPH oxidase NOX4 increased expression of cyclin-dependent kinase 1 (*CDK1*) and cell division cycle 25C/M-phase inducer phosphatase 3 (*CDC25c*), both of which promote cell cycling, anchorage-independent growth, and melanoma tumorigenesis *in vivo* (389).

Based on the previous observations, the growth stimulatory function of NADPH oxidase NOX4 is not limited to hematopoietic cells, but NADPH oxidase NOX4 can support also renal tumorigenesis *via* increased nuclear accumulation of hypoxia-inducible factor 2α (HIF-2α), therefore affecting phenotypic morphogenesis, colony formation, invasion, and *in vivo* tumor growth (114). In thyroid cancer models, NOX4 expression has been detected in papillary thyroid tumors and was shown to be stimulated by RAS and adenovirus E1A oncogenes, suggesting that NADPH oxidase NOX4 is a mediator of oncogene action (34, 374, 375). In addition, in a recent study, NOX4 expression was observed in thyroid cancer and in papillary thyroid cancer (PTC)-derived mesenchymal stem/stromal cells (MSCs) (275), thus indicating a paracrine role for NADPH oxidase NOX4 in thyroid function.

The tumor stroma microenvironment plays a crucial role in the initiation and development of tumors. The bidirectional paracrine effect activates tumor-associated fibroblasts to myofibroblasts, which then stimulate epithelial cell proliferation, migration, and metastasis (35). Transforming growth factor β (TGFβ) is a well-known regulator of cancer cell growth and stimulator of fibrotic reaction in several pathological conditions, such as renal fibrosis, liver cirrhosis, myocardial sclerosis, idiopathic pulmonary fibrosis, and desmoplastic reaction in advanced metastatic cancers. In general, TGFβ ligand binding to the TGFβR2/TGFβR1 heterodimer activates a cascade in which TGFβR2 is phosphorylated first, followed by the phosphorylation of TGFβR1.

The activated receptor dimer phosphorylates the SMAD2/SMAD3 dimer, allowing the association of SMAD4 into the complex. The SMAD complex then translocates to the nucleus, binds to DNA, and stimulates target gene expression. Interestingly, in the early phase of carcinogenesis, TGFβ functions as a tumor suppressor and as a tumor promoter at the end phase of carcinogenesis. This “TGFβ paradox” is caused by the modification of p53. Wild-type p53, which binds to the nuclear SMAD complex, inhibits growth together with tumor suppressor p63, whereas mutated p53, by binding to the SMAD complex, does not suppress growth and, additionally, inhibits p63 binding to SMAD complex (40).

Recently, TGFβ has been suggested to promote cancer cell migration through increased ROS production. The paracrine effect of human mammary MCF-7 cells has been demonstrated to activate the TGFβ-SMAD3 signal transduction pathway in mammary epithelial and stromal cells, inducing NOX4 expression and the consequent phosphorylation of focal adhesion kinase (FAK). FAK signaling downstream through the SRC and PI3K-AKT pathways is responsible for cell immobilization by attaching cells to ECM. Thus, the activation of FAK initiates local cancer cell migration and metastasis (27, 350).

**FIG. 4.**
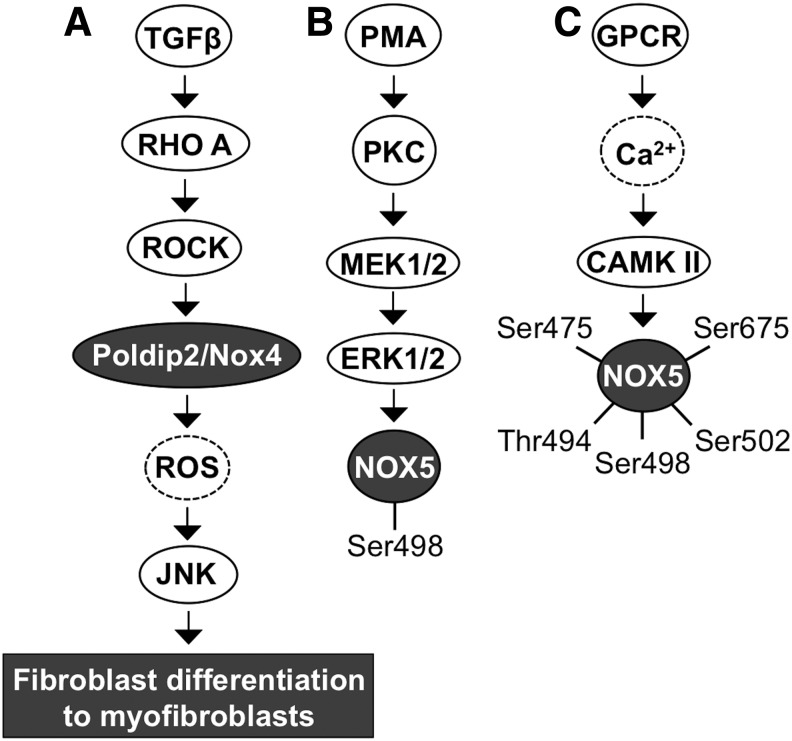
**NOX4 and NOX5 activation. (A)** TGFβ-driven activation of RHO-ROCK signaling increases NOX4 synthesis and consequent ROS production. **(B)** Mitogen stimulus caused by the PMA-activated PKC-MEK1/2-ERK1/2 signaling cascade stimulates NOX5 phosphorylation at Ser498. **(C)** GPCR activation increases Ca^2+^ influx, which then increases CAMKII and PKCα activation and consequently increases NOX5 phosphorylation at Ser475, Thr494, Ser498, Ser502, and Ser675. CAMKII, calcium/calmodulin-dependent kinase II; GPCR, G protein-coupled receptor; JNK, C-Jun N-terminal kinase; PMA, phorbol 12-myristate 13-acetate; ROS, reactive oxygen species; TGFβ, transforming growth factor β.

Fibroblast differentiation to myofibroblasts is the cornerstone in cancer progession. TGFβ contributes to fibrotic signaling by activating RHO small GTPase and the downstream RHO-associated kinase ROCK. Activation of ROCK phosphorylates POLDIP2, which increases *NOX4* gene and protein expression with consequent augmented ROS production. NADPH oxidase NOX4 has been demonstrated to be a mediator of TGFβ-RHO-ROCK-stimulated c-Jun N-terminal kinase (JNK) activation, which then increases myofibroblast differentiation-related gene expression—for example, insulin-like growth factor binding protein 3 (IGFBP3) and alpha smooth muscle cell acting expression—and morphological changes (216) (Fig. 4A).

## VII. NADPH Oxidase NOX5

There are two types of NOX5 genes; *NOX5-S* and *NOX5-L* (96). The latter has five splice variants; NOX5α, NOX5β, NOX5γ, NOX5δ, and NOX5ɛ, which functional significance is not completely characterized. Although all five splice variants are expressed in vascular endothelial and smooth muscle cells (20, 154), the expression of the splice variants shows also tissue-specific expression pattern: *NOX5α* is mainly expressed in the spleen, *NOX5β* is mainly expressed in the testis, and truncated *NOX5ɛ* is mainly expressed in esophageal cancer (15, 50, 94, 96). Isoforms have been suggested to contribute to coronary artery disease development, myocardial infarction, fetal ventricular septal defect, cancer, and irradiation-derived DNA damage in human primary fibroblasts and peripheral blood mononuclear cells (96, 376). In cells, NADPH oxidase NOX5 is located mostly in the endoplasmic reticulum where the enzyme mainly synthesizes O_2_^•−^ but has also been reported to produce H_2_O_2_ (10, 96, 139, 369).

Structurally, NADPH oxidase NOX5 is composed of six transmembrane domains carrying two heme molecules, a cytoplasmic N-terminus domain carrying four Ca^2+^ binding sites, and a cytoplasmic C-terminus domain carrying NADPH and FAD binding sites (50). On activation by Ca^2+^ binding to the N-terminal domain, the C-terminal domain-bound NADPH releases electrons to FAD and further to heme molecules, resulting in O_2_^•−^ production (96). Although NOX5 isoform activation is dependent on Ca^2+^, it is independent of the p40^phox^, p47^phox^, p67^phox^, and p22^phox^ cytosolic subunits. Ca^2+^ binding to N-terminal EF motif results in conformational changes, enabling the interaction between the EF motif and catalytic domain of the NOX5 subunit of NADPH oxidase (16, 48, 96).

Ca^2+^-cAMP response element-binding protein (CREB)- and Ca^2+^-PAF-ERK1/2-phospholipase A2 (PLA_2_)-STAT5-mediated signaling stimulate *NOX5* mRNA synthesis. Ca^2+^-dependent activation of PKCα and PKCɛ causes the phosphorylation of NOX5 at Ser490, Thr494, and Ser498. Interestingly, PKCδ seems to inhibit the production of NADPH oxidase NOX5-derived O_2_^•−^. However, the underlying mechanism causing the different effects of PKC isoforms is not well characterized but could be related to the regulation of Ca^2+^ influx or activation of Ca^2+^-related kinases.

PMA, demonstrated to activate MEK1/2-ERK1/2 kinase-driven phosphorylation of NOX5 at Ser498 without involving Ca^2+^ signaling, is necessary but not sufficient for NADPH oxidase NOX5 O_2_^•−^ production, thereby suggesting the involvement of other signaling routes (Fig. 4B). Indeed, direct interaction between calcium/calmodulin-dependent kinase II (CAMKII) and NOX5 results in NOX5 phosphorylation at Ser475, Ser498, Ser502, Ser675, and Thr494 (48, 94, 271, 272) (Fig. 4C).

### A. NADPH oxidase NOX5 in tumorigenesis

In gastroesophageal cancer, the activation of truncated form of NOX5, NOX5ɛ, is achieved by phosphatidylinositol-specific phospholipase C (PI-PLC), which releases IP3 and DAG. Interestingly, the signaling is mediated through ERK2, but not through ERK1, leading to full NOX5ɛ activation and ROS production (138). Another mechanism underlying how NOX5ɛ is activated in gastroesophageal cancer involves an increased intracellular Ca^2+^ concentration that activates small GTPase RHO and the downstream kinase ROCK2. ROCK2, but not ROCK1, then increases NOX5ɛ expression and H_2_O_2_ production (139).

Other signaling routes that upregulate and activate NOX5 are initiated by acid conditions or the IL-2 inflammatory cytokines, which activate the PAF-MEK-ERK-cytosolic phospholipase A_2_ (cPLA_2_)-JAK-STAT5-NOX5α/ɛ cascade in Barrett's esophageal adenocarcinoma cells and in human T cell leukemia virus type 1 (HTLV-1) transformed adult T cell leukemia cells (328, 331). Thereby, the signal transduction pathways connected to the activation of NADPH oxidase NOX5 corroborate the role of the oxidase in growth stimulation. Indeed, RNA interference (RNAi) silencing of NOX5α and the use of O_2_^•−^-neutralizing N-acetyl cysteine (NAC) and diphenyleniodonium (DPI) inhibited prostate cancer and leukemia cell proliferation *in vitro* and tumorigenesis *in vivo*, demonstrating NOX5α-driven cancer cell growth (136, 328, 331). NOX5 has been further shown to support growth and cell proliferation *via* the PDGF-JAK2-STAT pathway in vascular smooth muscle cells, *via* the NFκB-COX2-PGE_2_ pathway in esophageal adenocarcinoma cells, and *via* the SHP2-tyrosine PO4 pathway in hairy cell leukemia (96, 154).

## VIII. SOD1–3 Family

The SOD family consists of three isotypes: CuZnSOD (SOD1), MnSOD (SOD2), and EC-SOD (SOD3). SOD1 was first discovered in 1938 from bovine blood (hemocuprein) and from the liver (hepatocuprein) by Mann and Keilin (217), and in 1957 from the brain (cerebrocuprein) by Porter and Folch (290). McCord and Fridovich identified the discovered protein as an enzyme at the end of 1960s when they demonstrated the dismutase reaction and named the protein CuZnSOD (230). The second isotype, MnSOD, of the family was discovered in 1973 by Zimmermann *et al.* from mitochondria (399), and the third isotype, EC-SOD, was discovered by Marklund *et al.* from the extracellular space (222, 224).

SOD1, SOD2, and SOD3 catalyze the O_2_^•−^ conversion to H_2_O_2_ in two half reactions in which active center Cu^2+^ is first reduced to Cu^+^ and is then oxidized back to Cu^2+^ in a reduction/oxidation (redox) reaction (Fig. 1B). During the first half reaction, the oxidized form of the enzyme reacts with O_2_^•−^, releasing molecular oxygen. In the second half reaction, the reduced form of the enzyme reacts with the second O_2_^•−^ and with two protons (H^+^) releasing H_2_O_2_.

In the dismutation reaction, the negatively charged substrate O_2_^•−^ is guided into the positively charged channel of the enzyme by the electrostatic field created by the His-61, Glu-119, Lys-120, Glu130, Glu-131, and Lys-134 network. Once positively charged lysine amino acids of the network have attracted the substrate into the channel, Arg-141 further directs O_2_^•−^ into the copper-containing active center where the redox reaction of copper occurs. In the first half-reaction, His-61 disassociates from reduced copper and is forced out of the planar structure connecting the His-61 imidazole ring, copper, and zinc, forming a more tetrahedral structure. The geometrical change of the enzyme enables the second half reaction to occur (103).

## IX. Copper Zinc SOD, CuZnSOD, SOD1

**FIG. 5.**
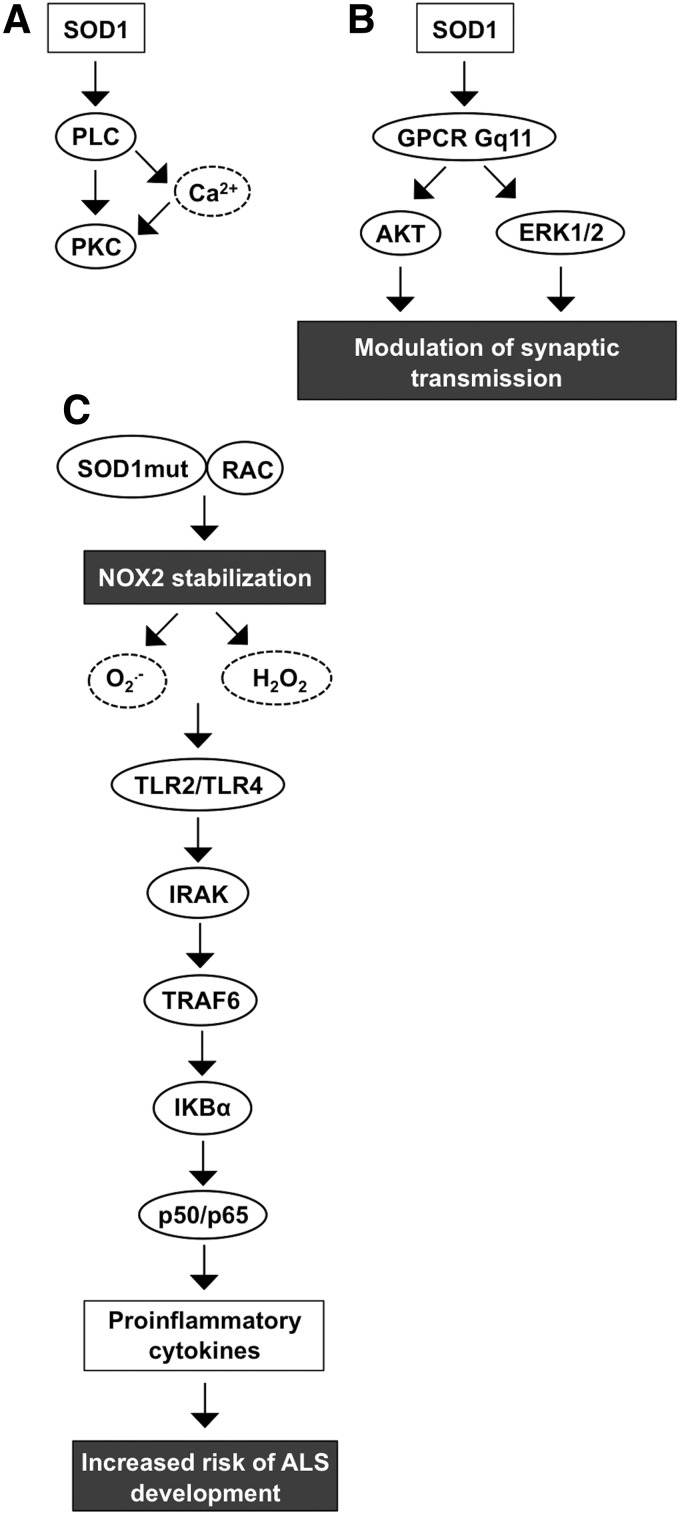
**SOD1 signal transduction. (A)** SOD1 activates PLC-Ca^2+^-PKC signaling. **(B)** Activation of GPCR Gq11 by SOD1 stimulates AKT and ERK1/2 signaling modulating synaptic transcriptions. **(C)** Mutant SOD1 binds to small GTPase RAC in an redox-insensitive manner causing NADPH oxidase NOX2 stabilization and increased proinflammatory cytokine production through TLR-IRAK-TRAF6 signal transduction. Increased cytokine production is a risk factor in ALS development. ALS, amyotrophic lateral sclerosis; IRAK, interleukin-1 receptor-associated kinase; TNF, tumor necrosis factor; TRAF6, TNF receptor-associated factor 6.

Cytosolic SOD1 is a 32-kDa homodimer formed from two catalytically active nondisulfide-linked subunits. Although the stimulation of *SOD1* gene expression is not completely characterized, the promoter region has binding sites for specificity protein 1 (SP1), early growth response 1 (EGR-1), and activator protein 1 (AP1) along with upstream enhancer region-located binding sites for NFκB, nuclear factor (erythroid-derived 2)-like 2 (NRF2), and CCAAT enhancer-binding protein (C/EBP), possibly indicating putative transcription regulatory mechanisms (172, 276, 320). SOD1 has been shown to increase PLC-PKC signal transduction that opens voltage-gated Ca^2+^ channels, allowing Ca^2+^ influx and causing an increased intracellular Ca^2+^ concentration in human neuroblastoma cells (247) (Fig. 5A). Other neuronal signal pathways that SOD1 activates include muscarinic M1 acetylcholine receptor coupled to Gq_11_-ERK1/2 and AKT kinase cascades, modulating synaptic transmission, thus suggesting a function for the enzyme in the neuronal microenvironment (69, 246) (Fig. 5B).

SOD1 has been connected to the development of amyotrophic lateral sclerosis (ALS), cancer, ischemia, and altered glucose metabolism (274, 297, 302). The evidence connecting SOD1 and ALS is based on observations suggesting a high number of mutations that affect the ability of the enzyme to increase the risk of ALS development. Hitherto, there are at least 170 known mutations that are linked to ALS. Mice carrying mutant *SOD1* show up to 50-fold reduced affinity for zinc located at the active center of the enzyme (131). Therefore, mutations affecting the stability of the SOD1 active center disrupt the normal activity of the enzyme and may even convert the antioxidant function, thereby increasing the oxidative stress (327).

Under reducing conditions, mutant SOD1 can regulate NOX2 activation by stabilizing RAC1 through direct binding to the small GTPase in a redox-insensitive manner, enhancing the NOX2 complex assembly and ROS production. Both wild-type and mutant SOD1 interact with RAC1, maintaining the small GTPase in the active GTP-bound form. Importantly, the interaction between wild-type SOD1 and RAC1 is disrupted at increased H_2_O_2_ concentrations, thus allowing the hydrolysis of GTP from RAC1, whereas mutant SOD1 lacks redox sensitivity, maintaining the active RAC1-GTP complex, NADPH oxidase NOX2 oxidative burst, and increased production of ROS (120, 157).

Increased O_2_^•−^ and H_2_O_2_ production activates Toll-like receptor 2 (TLR2) and TLR4 signal transduction through IL-1 receptor-associated kinase (IRAK), BH3 domain-only, tumor necrosis factor (TNF) receptor-associated factor 6 (TRAF6), and the IκBα-p50/p65 pathway, eventually increasing the proinflammatory cytokine expression characteristic of ALS (174) (Fig. 5C).

### A. SOD1 in tumorigenesis

SOD1 has a diverse effect on cancer cell signal transduction, growth, and survival. Inhibition of SOD1 with the ATN-224 small-molecule inhibitor has been suggested to inhibit epidermal growth factor (EGF)- and insulin growth factor (IGF)-stimulated mitogen signal transduction through ERK1/2 kinases. Mechanistically, the inhibition of SOD1 maintains the concentration of H_2_O_2_ at levels that are not adequate to inhibit PTPs, thereby allowing the PTP-mediated inactivation of RTK phosphorylation and attenuation of corresponding downstream signal transduction (158). Inhibition of SOD1 by ATN-224 conceivably increases the intracellular O_2_^•−^ concentration, which, surprisingly, leads to the inhibition of glutathione peroxidase activity with a consequently increased lethal level of intracellular H_2_O_2_ concentration.

H_2_O_2_ induces the expression of proapoptotic Bcl-2 interacting mediator of cell death (BIM) and BCL2 binding component 3 (PUMA), and phosphorylation of p38MAPK, which then causes decreased antiapoptotic factor myeloid cell leukemia 1 (MCL1) expression. Consequently, ATN-224-treated cancer cells enter programmed caspase-mediated apoptosis *in vitro* and *in vivo* (110, 158) suggesting SOD1 as a tumor promoter and a potential novel target for cancer therapy.

SOD1 has been shown to attenuate cellular respiration by increasing aerobic glycolysis in glucose metabolism. In general, glycolysis, which occurs in the cytoplasm, is one of the main ATP synthesis mechanisms in cells together with mitochondrial oxidative phosphorylation. In normal cell glycolysis, glucose is metabolized to pyruvate, which is further metabolized to CO_2_ in the mitochondrial oxidative phosphorylation reaction cascade in the presence of O_2_. In the absence of O_2_, pyruvate is metabolized in anaerobic glycolysis, resulting in markedly lower levels of ATP. In cancer cells that have increased glycolysis, and thus increased microenvironmental acidosis, pyruvate is mostly metabolized to lactate in aerobic glycolysis (309). The study of the contribution of SOD1 to glycolysis suggested that *Saccharomyces cerevisiae* yeast casein kinase 1 gamma (CK1γ) homologues Yck1p and Yck2p are stabilized by SOD1 dismutase function.

Functionally, SOD1 utilizes yeast Yno1p NAPDH oxidase-like protein produced O_2_^•−^ to increase the cellular H_2_O_2_ concentration in human cells (297), suggesting that mammalian NOX enzymes and SOD1 are signaling partners in the modification of glucose metabolism in cellular respiration increasing aerobic glycolysis. Therefore, SOD1 supports cancer cell growth by maintaining proliferative signal transduction, protecting cells from ROS-derived induction of apoptosis and inducing the metabolic switch from normal respiration to aerobic glycolysis.

## X. Manganese SOD, MnSOD, SOD2

Tetrameric manganese Mn-II coenzyme containing SOD2 is located almost exclusively in mitochondria, where it converts respiratory chain-produced O_2_^•−^ into H_2_O_2_. Each SOD2 monomer contains seven α-helices, three antiparallel β-sheets, and seven connecting structures. The manganese-containing site of the enzyme is associated with N-terminal helical hairpin domain and C-terminal α-helix/β-sheet structures. The importance of the enzyme to cellular viability has been demonstrated by studies showing the lethal phenotype of SOD2-knockout mice (201), underlining the critical role of the O_2_^•−^-H_2_O_2_ balance in the mitochondria. The enzyme expression is induced by a high number of factors, including oxidative stress, cytokines, and transcription factors, such as NFκB, SP1, CREB, and different members of the forkhead box family (FOXO) (58, 218, 385).

Inhibition of the NFκB signal transduction pathway by the kinesin spindle protein inhibitor SB715992 (1 n*M* concentration) in human multiple myeloma cells results in reduced SOD2 expression and induces cell death in 24 h (335), therefore confirming the role of NFκB in SOD2 stimulation and suggesting a growth-supportive role for the enzyme. Indeed, overexpression of *miR146a* downregulated *SOD2*, reduced human epithelial ovarian cancer cell proliferation, increased apoptosis, and increased sensitivity to chemotherapy (67). Similar data were obtained for Bcl-2-overexpressing metastatic B16 melanoma cells treated with *SOD2* and *Bcl2* antisense oligonucleotides. The treatment significantly reduced the number and viability of the cells and increased apoptosis, necrosis, and sensitivity to chemotherapy in the presence of tumor necrosis factor alpha (TNFα) (22).

Studies investigating the role of SOD2 in radiation protection following irradiation cancer therapy in human neuroblastoma, Ewing sarcoma, breast cancer, bladder cancer, colon cancer, prostate cancer, and lung cancer cells demonstrated that low-dose irradiation (2–100 cGY) increased NFκB activation and consequently increased SOD2 expression, as well as the survival/clonal expansion of cells (11). Hence, based on these data, SOD2 may represent a potential target for combination cancer therapy treatments.

### A. SOD2 in tumorigenesis

**FIG. 6.**
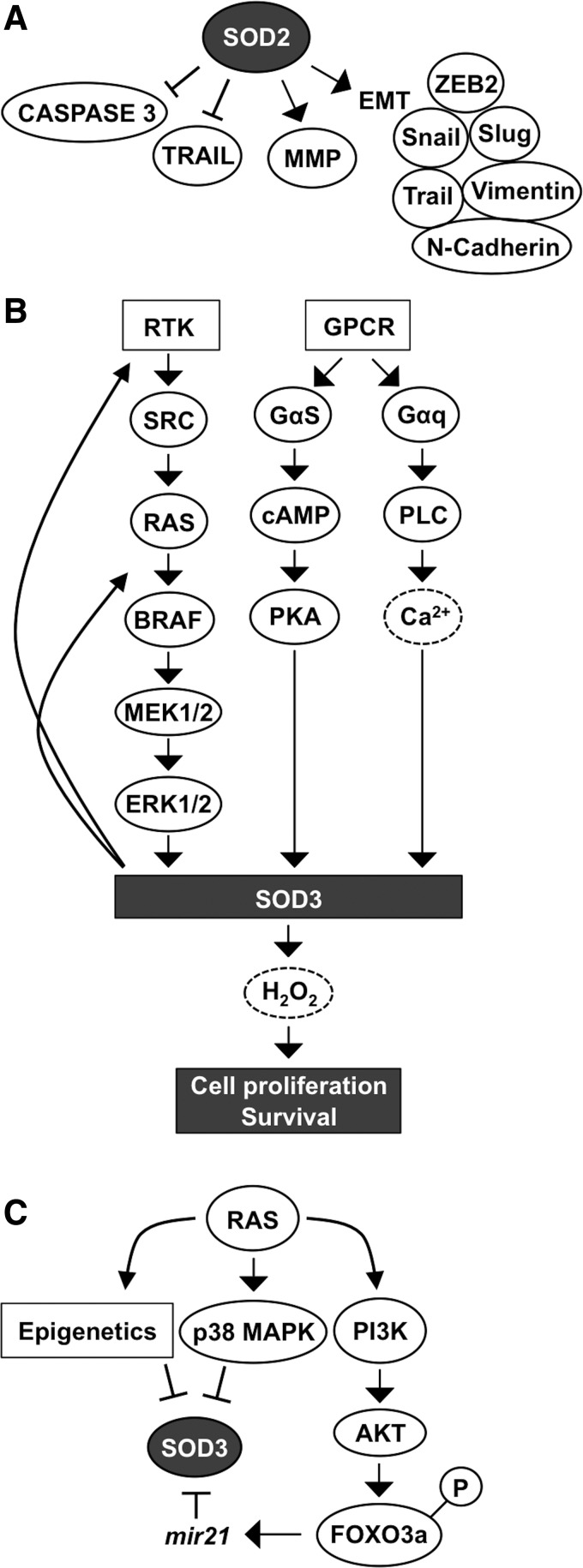
**SOD2 and SOD3 in cell metastasis and in cell survival. (A)** SOD2 promotes metastatic signaling molecule activation and inhibits apoptosis by downregulating caspase 3 and TRAIL, and by upregulating EMT mediating proteins and MMPs. **(B)** RTK-SRC-RAS-ERK1/2, GPCR-cAMP-PKA, and GPCR-PLC-Ca^2+^ signaling activate SOD3 production, thereby increasing cell proliferation and survival. Positive feedback signaling increases RTK phosphorylation and RAS GTP loading, thus maintaining activity of the RAS-ERK1/2 cascade. **(C)** RAS controls SOD3 expression through epigenetics, by p38MAPK activation, and by the PI3K-AKT-FOXO3a pathway. PI3K-AKT activation causes phosphorylation of the transcription factor, FOXO3a, resulting in its transfer from the nucleus to the cytoplasm, thereby increasing *mir21* synthesis, which targets *SOD3* mRNA. EMT, epithelial/mesenchymal transition; FOXO, forkhead box family; PI3K, phosphatidylinositol-4,5-bisphosphate 3-kinase; TRAIL, TNF-related apoptosis-inducing ligand.

Early reports concerning SOD2 function in carcinogenesis showed a correlation between decreased SOD2 expression at the early initial stage of carcinogenesis and suggested a tumor suppressor role for the enzyme (59, 262, 289, 397). However, SOD2 expression has been shown to increase in late-stage aggressive and metastatic cancers and cell models (61, 125, 142, 161, 165, 175, 378, 384), indicating the importance of the enzyme for the progression of cancer. The conclusion is corroborated by the data demonstrating SOD2-driven inhibition of apoptosis and increased mRNA expression of MMPs *MMP1*, *MMP2*, and *MMP9* in several cancers and cancer cell lines, such as HT1080 fibrosarcoma cells, MCF-7 breast cancer cells, 253J bladder carcinoma cells, and 253J-BV metastatic bladder cancer cells (61, 129, 258, 395) (Fig. 6A).

Decreased apoptosis correlating with increased SOD2 expression could be mediated by the stabilization of the mitochondrial membrane, decreased caspase 3 activation, and inhibition of TNF-related apoptosis-inducing ligand (TRAIL) (244). Increased SOD2-derived MMP expression was demonstrated to correlate with increased migration, increased invasion, and increased metastasis (Fig. 6A). The exact mechanism underlying how SOD2 increases *MMP* gene expression and the metastatic potential of the cells has not been completely characterized, but it could be related to the modification of *MMP* gene promoter region activity, manipulation of phosphatase and tensin homologue (PTEN), protein tyrosine phosphatase, nonreceptor type 12 (PTPN12), and MAP kinase phosphatase activity, and the maintenance of mitochondrial integrity and metabolic capacity (125, 126).

In addition, increased SOD2 expression is correlated with an increased epithelial–mesenchymal transition (EMT) score in different breast cancer subtypes, whereas the RNAi silencing of SOD2 decreases EMT-related protein (VIMENTIN, β*-*CATENIN, SLUG, N-CADHERIN, TWIST, zinc finger E-box binding homeobox 2 [ZEB2], and SNAIL) expression (126, 165, 175, 207), strongly suggesting a stimulatory role for SOD2 in cancer cell migration and metastasis.

The enhancer function of SOD2 in cellular migration and metastasis is further supported by data demonstrating the correlation of SOD2 expression and phosphorylation of breast cancer anti-estrogen resistance protein 1 (BCAR1), also known as p130Cas. The overexpression of SOD2 increases BCAR1 phosphorylation and membrane recruitment, evidently causing the oxidation inactivation of PTEN (62, 127). PTEN is a tumor suppressor gene that converts PIP3 to PIP2, counteracting the conversion of PIP2 to PIP3 by PI3K. Decreased PTEN activation allows the accumulation of PIP3, thereby maintaining downstream AKT kinase in the active state, which plays a fundamental role in the regulation of growth, proliferation, apoptosis, angiogenesis, and metabolic activities of the cells.

BCAR1 is a docking protein that has several protein–protein interaction domains with a prominent role in cell adhesion and initiation of cell migration, creating the link between increased SOD2 expression and cellular cytoskeletal modification. The amino terminal SH3 domain of BCAR1 has been shown to interact with FAK and protein tyrosine kinase 2β (PTK2β), also known as the PYK2 nonreceptor tyrosine kinase of the FAK family, inducing their activation. The BCAR1 and PTK2β interaction with FAK induces adherent junction disassembly, modifications of focal adhesions, and cellular cytoskeletal structure. More importantly, FAK-BCAR1 signaling activates the small GTPase RAC1 and CDC42 (a member of the RHO small GTPase family) with the subsequent induction of membrane protrusions and cell migration (63, 127).

Recently, it was demonstrated that PKC-ERK1/2 signal transduction upregulates mitochondrial SOD2 expression in response to glucose starvation in lung carcinoma cells (170). Indeed, increased SOD2 expression parallels with glycolytic metabolism, especially in aggressive metastatic cancers, causing the activation of 5′ adenosine monophosphate-activated kinase (AMPK), which further stimulates glycolysis. In fact, a recent study demonstrated SOD2-driven increased AMPK phosphorylation at Thr172 in breast, colon, and prostate cancers, suggesting the involvement of SOD2 in the glycolytic switch in cancer progression that may represent a mechanism to regulate the Warburg effect. Interestingly, the data demonstrate the interaction of SOD2-derived cell metabolism *via* AMPK signaling and NADPH oxidase-derived ROS production.

In addition to modifications of glycolytic metabolism, SOD2 signal transduction *via* AMPK inhibits apoptosis and increases drug resistance and colony formation *in vitro* in soft agar, all characteristics of late-stage cancers (121). The *in vitro* results are further corroborated by clinical data, demonstrating the correlation between increased expression, poor survival, disease relapse, and metastasis in head and neck squamous cell carcinoma, pancreatic cancer, gastric cancer, and colorectal carcinoma (84, 165, 288).

## XI. Extracellular SOD, EC-SOD, SOD3

Secreted extracellular SOD is the latest member of the mammalian SOD isoform family. The enzyme is composed of two covalently associated dimers, which form a tetrameric protein except for rat SOD3, which has a dimeric structure (38, 379). Each SOD3 subunit has four domains: (i) secretion domain; (ii) amino terminal glycosylated (Asn-89) domain, which increases the solubility of the enzyme; (iii) copper- and zinc-containing active center domain; and (iv) C-terminal heparan sulfate binding domain that contains a proteolysis-sensitive segment causing the sequential degradation of the enzyme with consequent loss of binding capacity to cell membranes.

Based on the cell membrane heparan sulfate affinity, SOD3 can be classified into three (A, B, and C) or five (I–V) subgroups: the intact secreted C-form has all four C-terminal ends intact and is referred to as classes IV and V; the B-form has reduced affinity due to protease degradation and is referred to as classes II and III; and the A-form is soluble lacking C-terminal heparan sulfate binding domains and is referred to as class I (4, 162). The recent observations suggest that the functional response of the cell membrane-bound intact C-form SOD3 on cells and tissues is mediated in an autocrine and a paracrine manner (2, 192, 195, 275). A number of reports have demonstrated that SOD3 has antioxidative, antiapoptotic, anti-inflammatory, and growth-promoting characteristics in tissue injury models, in genetically modified mice, and in cancer models (39, 91, 194, 195, 197, 223). Indeed, SOD3 was first identified as a therapeutic enzyme being able to inhibit efficiently liver damage in a paracetamol intoxication model, cardiovascular damage in a reperfusion model, and neointima growth in a restenosis model and only recently shown to promote unwanted growth in cancer models (39, 184, 189, 190, 192, 194, 195, 197, 332).

The signal transduction pathways RAS-BRAF-MEK1/2-ERK1/2, GPCR-Gαs-cAMP-PKA, and GPCR-Gαq-PLC-Ca^2+^, which increase the enzyme levels (34, 93, 184, 197), and p38MAPK, which inhibits the expression (3), are the most prominent signaling cascades regulating SOD3 synthesis. Interestingly, increased SOD3 expression has a stimulatory effect on RAS activation by GTP loading, suggesting a positive feedback loop: SOD3 increases RAS activation, which induces downstream signaling, causing increased SOD3 production (Fig. 6C) (190, 197). More interestingly, the positive feedback loop, which maintains the mitogenic RAS-ERK1/2 signal pathway active, could potentially represent a mechanism underlying how SOD3 mediates tissue injury healing.

RAS is fundamentally connected to SOD3 signaling by regulating *SOD3* mRNA synthesis through the MEK1/2-ERK1/2 cascade and by inhibiting gene expression through p38 MAPK phosphorylation, methylation, and acetylation. In addition, RAS activates the PI3K-AKT pathway, which induces FOXO3a inactivation by phosphorylation, with consequent increased *mir21* synthesis, and targets *SOD3* mRNA, interfering gene expression (33, 185, 396) (Fig. 6C). Although SOD3 expression at physiological levels supports growth, the enzyme is paradoxically downregulated in certain cancers. The gradual inhibition of SOD3 expression correlates with the oncogene activation level involving small GTPase regulatory genes, epigenetic regulation of gene expression, *mir21* stimulation by AKT-FOXO3a phosphorylation, and p38MAPK signaling (33, 184, 190, 262, 393, 394, 396) (Fig. 6C).

The fine-tuning of RAS downstream signaling is controlled by RAS GTPase regulatory genes guanine nucleotide exchange factor (GEF), GAP, and guanine nucleotide disassociation inhibitor (GDI), which have a balancing effect on SOD3 expression levels. Moderately 6- to 10-fold increased RAS activation stimulates *SOD3* mRNA synthesis until there is a sudden decrease in the enzyme mRNA production at more aberrant RAS activation levels. Simultaneously with decreased *SOD3* mRNA expression, there is an inverse correlation with increased *mir21* expression and increased p38 MAPK phosphorylation, both induced by RAS (33). The microRNA *mir21* has been shown to bind to 3′ untranslated region (UTR) in *SOD3* mRNA, thus causing the degradation of the messenger (396). In advanced cancers, RAS-induced epigenetic methylation and acetylation have been shown to contribute to more pronounce silencing of SOD3. Therefore, the regulation of SOD3 expression can be divided into reversible regulation by small GTPase regulatory genes, *mir21*, or p38MAPK and irreversible epigenetic silencing by methylation and acetylation (33).

### A. SOD3 in tumorigenesis

Similar to SOD2, early studies have suggested that SOD3 is a tumor suppressor gene downregulated in cancer. Various works using transgenic SOD3 mice or adenovirus gene transfer techniques have demonstrated the inhibitory function of SOD3 on cancer cell proliferation and tumor growth. In all these early studies, the expression level of the enzyme was highly above the physiological levels observed in tissues and cells (346, 347, 377) causing aberrant growth signal affecting small GTPases and β-CATENIN signaling (190). More recent works have demonstrated a dose-dependent growth response for the enzyme, clarifying the controversy between recently and previously published data and demonstrating that, at the physiological level or at moderately increased level, SOD3 supports cell proliferation and cell survival, and reduces apoptosis by regulating RAS activation (39, 189, 190, 197, 368).

Importantly, increased SOD3 expression results in increased phosphorylation of various growth-related cell membrane RTKs and RTK-associated signaling molecules, such as SRC proto-oncogene family members, regardless of the SOD3 expression level. It is not quite clear how enzyme overexpression increases RTK phosphorylation, but it could be mediated by the ability of H_2_O_2_ to oxidatively inactivate PTPs by modifying the catalytic site cysteine amino acids, thereby causing persistent RTK activation as a response of ligand binding to the receptor (266, 267, 354). Therefore, SOD3 itself would not be a growth factor, but the enzyme, by producing H_2_O_2_, could represent a regulator of growth-related signaling molecule activity.

**FIG. 7.**
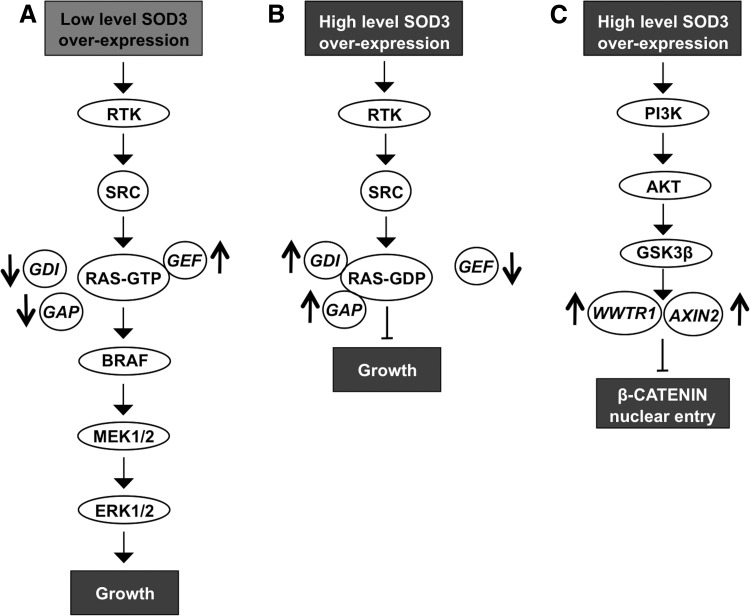
**SOD3 regulation of signal transduction. (A)** Low level of SOD3 expression increases RTK and SRC phosphorylation and allows signaling through small GTPase RAS by increasing GEF expression and decreasing GAP and GDI expression. **(B)** High level of SOD3 expression increases RTK and SRC phosphorylation but inactivates small GPTase RAS by decreasing GEF expression and increasing GAP and GDI expression. Thereby, the signal does not proceed to mitogenic pathway. **(C)** High level of SOD3 expression increases WWTR1 and AXIN2 expression inhibiting β-CATENIN nuclear entry, which then attenuates growth. AXIN2, axis inhibition protein 2; GDI, guanine nucleotide disassociation inhibitor; GEF, guanine nucleotide exchange factor; WWTR1, WW domain containing transcription regulator 1.

The regulatory role of SOD3 in signaling is further corroborated by data suggesting SOD3 dose-dependent activation of small GTPases. At high SOD3 levels, the expression of RAS regulatory gene *GEF* mRNA synthesis is downregulated, whereas *GAP*s and *GDI*s, which maintain RAS in the inactive GDP-bound form in the cytosolic compartment, are increased. Thus, SOD3-driven modification of small GTPase regulatory gene expression inhibits the progression of signal transduction downstream of RAS. Moderately increased (∼2- to 4-fold increased enzyme activation level and 10- to 15-fold increased mRNA level) SOD3 expression stimulates GTP loading to small GTPases, thus allowing cell membrane signal transduction to pass RAS to the downstream signaling network (190, 197) (Fig. 7A, B).

Other control checkpoints for signaling observed at high SOD3 expression levels include, for example, β-CATENIN entry into the nucleus. High SOD3 concentration increases the expression of *WWTR1* (WW domain containing transcription regulator 1) and *AXIN2* genes, which maintain β-CATENIN in the cytoplasm. The growth regulatory role of SOD3 is further strengthened by KEGG and GO functional pathway analysis, suggesting the highest impact on growth and proliferation signaling (190, 197) (Fig. 7C).

Although the expression of the enzyme is gradually downregulated in thyroid cancers and thyroid cancer cell lines, a recent article demonstrated increased *SOD3* mRNA synthesis in PTC MSCs, therefore suggesting an autocrine/paracrine switch in SOD3 production (39, 184, 275). Increased *SOD3* expression in tumor stroma MSCs stimulated thyroid cancer cell proliferation but decreased cancer cell migration, indicating that SOD3 may reduce the intratumoral affinity of cancer cells and allows them to migrate locally toward peritumoral regions (275). Previously, SOD3 has been shown to reduce cellular migration by downregulating inflammatory cytokines and intracellular adhesion molecule 1 (ICAM-1), vascular cell adhesion molecule 1 (VCAM-1), and E-selectin and P-selectin adhesion (198) molecules, which are involved in cell/cell interaction structures, such as in tight junctions. Therefore, the data may suggest that, at the late phase of carcinogenesis, the autocrine/paracrine switch maintains SOD3-driven growth support but, at the same time, releases epithelial cancer cells to migrate toward normal healthy tissue (275).

## XII. Interaction of NOX1–5- and SOD1–3-Associated Signaling

### A. GPCR signaling

**FIG. 8.**
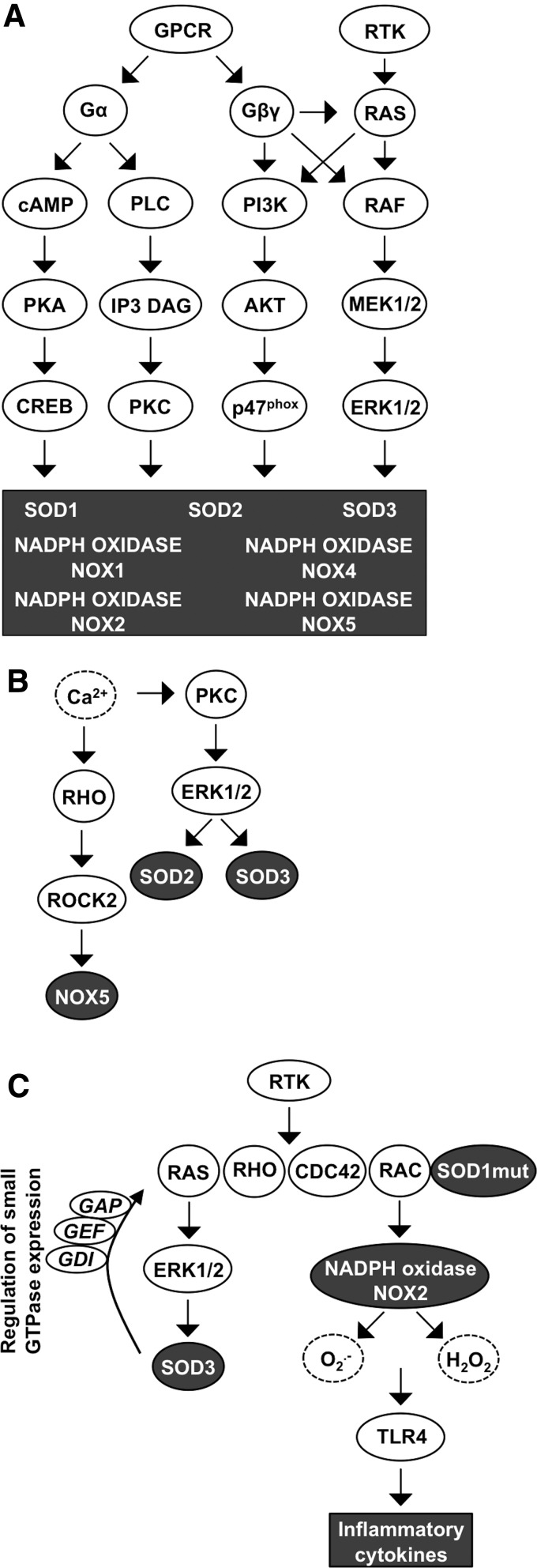
**Interaction of GPCR, Ca^2+^, RTK, and small GTPase signaling affecting redox gene expression. (A)** GPCR Gα and Gβγ activation increases signaling through cAMP, PLC, small GTPases, PI3K-AKT, RAS, and RAF, which are among the redox-linked signal transduction molecules, thereby stimulating expression of SOD1–3 and NADPH oxidases NOX1, −2, −4, and −5. RTK activation occurs at the level of RAF and PI3K signaling molecules. **(B)** Ca^2+^ increases small GTPase RHO-ROCK signaling that stimulates *NOX5* expression. Ca^2+^ activates also PKC-ERK1/2 pathway causing increased SOD2 and SOD3 production. **(C)** RTK and small GTPase activation stimulate ERK1/2 signal transduction that promotes SOD3 expression and formation of a regulatory loop affecting small GTPase activation. Mutant SOD1 binds to small GPTase RAC in a redox-insensitive manner causing continuous ROS production and inflammatory cytokine synthesis.

GPCRs could be the most important signaling elements that integrate NOX1–5 and SOD1–3 signal transduction. GPCRs form the largest family of cell membrane-associated signaling molecules consisting of over 800 members expressed in all cell and tissue types. GPCR is activated by many ligands representing peptides and proteins, lipids, amino acids and ions, biogenic amines, and a heterogenic group of substances, such as odorants, nucleotides, endorphins, and even light. GPCRs are integral cell membrane receptors that contain seven transmembrane α-helical regions and bind to a wide range of extracellular ligands. Ligand binding to GPCR initiates a conformational change in the receptor structure, allowing interaction with the heterotrimeric large G protein subunit α (α_s_, α_i_, α_q_, or α_12_) with consequent GDP to GTP catalysis activating the large G protein. On activation, the G protein subunit β (β_1–5_) and subunit γ (γ_1–14_) dissociate from Gα and function as a heterodimer stimulating PI3K, PLCβ, and, in some cases, also small GTPase RAS and RHO activation. Large G protein signaling activation continues until GTP is hydrolyzed from the Gα subunit, which then adheres back to the Gβγ dimer, thereby ending the downstream pathway activation (81, 221, 261) (Fig. 8A).

Characteristically, large Gα proteins are divided into different groups according to the large G protein they interact with: (i) Gα_i_ (adenylyl cyclase inhibitor) inhibits cAMP synthesis but activates the SRC proto-oncogene family and downstream MAPK growth, angiogenesis, and survival signaling; (ii) Gα_s_ subunit antagonizes Gα_i_ function-activating adenylyl cyclase and cAMP-dependent PKA activation that phosphorylates CREB transcription factor with the consequent increased expression of target genes involved in DNA repair, oncogenesis, and autophagy; (iii) Gα_q_ proteins activate PLC signaling that catalyzes the formation of IP3 and DAG, leading to the activation of PKC; and (iv) Gα_12_ downstream target molecule small GTPase RHO and RAC modify actin cytoskeleton reorganization and focal adhesions and are frequently involved in carcinogenesis (283).

GPCRs regulate essentially almost all normal cellular functions, such as blood pressure, by altering the heart rate, vascular resistance, and fluid/electrolyte balance. Regulation of the immune system activation by controlling immune cell chemotaxis, homing, activation, and target tissue recruitment is another important physiological response of GPCRs, as well as kidney sodium secretion, and bone mass and architecture modeling. Because ∼90% of GPCRs are expressed in the brain, their physiological function is foremost characterized in the neuronal environment (292). According to recent studies, transforming cells utilize the normal physiological functions of GPCRs to promote their autonomous proliferation, escape from immune recognition, and survive during metastatic invasion, intravasation, and extravasation (81, 221, 261).

GPCRs have been shown to mediate NOX4-derived ROS production in heart failure on ligand binding (Fig. 8A). Binding of the agonist to the receptor induces the phosphorylation of G-protein-coupled receptor kinase 2 (GRK2) and recruitment of β-arrestin, initiating downstream signaling and increasing NOX4 activity. Increased ROS production by NOX4 augments myocardial dysfunction and DNA damage, and initiates apoptotic cell death program in cardiac myocytes, thereby increasing the risk for chronic heart failure (348). In astrocytes, ATP ligand binding to purinoceptor 7 (P2X7) receptors, which represent P1 GPCRs, has been demonstrated to augment NOX4 expression and activation. Altered ROS production in microglia has a direct impact on the pro/anti-inflammatory role that cells may play in neurodegenerative diseases, such as Alzheimer's disease, Parkinson's disease, and in ALS (232).

Nucleotides, such as ATP, activate PY2 purinergic GPCRs, inducing PLC-cAMP-Ca^2+^ signal transduction, protecting astrocytes against ROS-derived damage. The interaction between GPCR and SODs is highlighted by the data suggesting increased *SOD2* and *SOD3* expression caused by the activation of purinergic P2Y GPCR and thyroid-stimulating hormone receptor (TSHR) class A GPCR. Mechanistically, ligand binding to GPCR class A and purinergic P2Y GPCR activates the Gα_s_-cAMP-PKA pathway and/or Gα_q_ PLC-Ca^2+^ signaling, increasing the expression of *SOD2* and *SOD3*, which then regulate normal thyrocyte proliferation and resistance against apoptosis (93, 184) (Fig. 8A).

Another unstudied connection between SOD3- and GPCR-mediated growth signaling comes from data demonstrating the regulation of regulator of G protein signaling 4 (*RGS4*) expression by SOD3 (190). The results observed in metastatic anaplastic thyroid cancer cells suggest a putative role for the enzyme in GPCR signaling, although currently there are no reports showing SOD3-driven regulation of large G protein signaling. SOD1 activation has been studied in astrocytes in which peptides, such as octadecaneuropeptide (ODN), can bind G_1/0_ GPCR, resulting in markedly increased *SOD1* expression already minutes after ligand binding. The signaling increasing SOD1 production is specifically channeled *via* PKA as its inhibitor, H89, has been reported to abrogate enzyme production (117) (Fig. 8A).

GPCR signaling activating small GTPase RAC is frequently associated with actin modification. Large Gα_12_ activation initiates cytoskeletal remodeling affecting focal adhesion integrity, thereby allowing cells to migrate locally (81, 221, 261). Similar to Gα_12_, noncanonical WNT (wingless-type MMTV integration site family) signaling also recruits RAC, as well as RHO and CDC42, inducing cytoskeletal reorganization. RHO, which signals through ROCK, regulates actin filament assembly to form mainly lamellipodia, whereas RAC and CDC42 promote actin polymerization at the cell periphery, constructing primarily filopodia (150, 228). Under certain conditions, GPCR and noncanonical WNT signaling stimulate the PI3K-AKT pathway, which are, as explained above, involved in p47^phox^ phosphorylation, initiating NAPDH NOX assembly (40, 137, 180). However, the activation of PI3K-AKT signaling is more thoroughly studied in the context of RTK phosphorylation that induces SRC proto-oncogene activation in addition to downstream ERK1/2 and p38MAPK signal transduction, all connected to redox signaling (Fig. 8A).

Chemokine receptors represent a class of GPCRs composed of ∼20 members that mediate various cellular events, such as cell migration and survival. CXCR4/SDF-1 interaction has been shown to mediate the homing and quiescence of hematopoietic stem cells (HSCs); however, in cancer, CXCR4-positive cells are more prone to metastasis (187). In prostate cancer cells, SOD1 has been reported to make a direct contact with the first intracellular loop of CXCR4 in an SDF-1 ligand binding-dependent manner. Interaction of SOD1 with CXCR4 phosphorylates AKT, inhibiting the function of proapoptotic proteins, such as Bcl-2-associated death promoter (BAD), and activating antiapoptotic signal transduction (392).

### B. Ca^2+^ signaling

The most noticeable factor connecting redox enzymes to cellular functions is the reciprocal signaling of ROS and Ca^2+^, which coordinates cellular functions in cancer, such as metabolism and gene expression, cell fate, apoptosis or growth, in a dose-dependent manner. The biological response of Ca^2+^ is caused by direct binding to target proteins without catabolic or anabolic modifications (112). Interestingly, cell adhesion to the vascular wall is enhanced by mitochondrial ROS production stimulated by GPCR-Ca^2+^ signaling.

Mechanistically, ligand binding to GPCR stimulates the interaction of IP3 to its mitochondrial membrane receptor InsP3R, increasing the mitochondrial matrix Ca^2+^ concentration. The released Ca^2+^ then stimulates mitochondrial NADH and O_2_^•−^ production as a part of ATP generation (123). In support of this, recent data demonstrate that increased O_2_^•−^ promotes NFκB signaling, which stimulates ICAM-1 expression, which is critical for leukocyte adhesion to vascular endothelial cells. In addition, ICAM-1 expression is inhibited by the SOD mimetic MnTBAP (123) and by increased expression of SOD3 (198), corroborating conclusions that cell adhesion molecule expression could be O_2_^•−^ dependent.

As mentioned above, Ca^2+^ functions as a mediator of GPCR signal transduction, influencing the redox enzyme expression level and mediating the ROS-derived message into the cellular signal transduction network. ROS, especially H_2_O_2_, directly control the intracellular Ca^2+^ concentration by oxidizing cysteine residues within Ca^2+^ channels or within Ca^2+^ channel activators, such as stroma interacting molecule-1 (STIM1), affecting protein conformation and activity (128, 199). Similar to mitochondrial Ca^2+^ influx, cell membrane GPCR-derived IP3 can interact with IP3R at the endoplasmic reticulum, which is one of the major intracellular Ca^2+^ storage sites in addition to sarcolemma, allowing Ca^2+^ flow into the cytoplasm (78). Modifications in the intracellular Ca^2+^ concentrations consecutively activate downstream signaling, stimulating the PKC pathway that causes increased NADPH NOX and SOD activation (128) (Fig. 8B).

Although Ca^2+^ does not directly activate NOX1 and NOX3, it is involved in the stimulation of upstream small GTPase, thereby increasing signal transduction and leading to NADPH NOX1 and NADPH NOX3 complex activation (19, 29). GPCR signal transduction inducing the IP3 pathway and increased intracellular Ca^2+^ concentration have been shown to activate the small GTPase RHO-ROCK2 cascade that sequentially increases *NOX5* mRNA production (29). NAPDH NOX5-derived O_2_^•−^ production is directly dependent on Ca^2+^, which induces a conformational change connecting the N-terminal regulatory site with the C-terminal catalytic region. The change in the three-dimensional EF motif structure could be caused by the reorganization of Cys109 residues, allowing the N-terminal EF motif to contact the catalytic C-terminus that contains binding sites for FAD and NADPH (16) (Fig. 8B).

In ALS models, Ca^2+^ has been demonstrated to cause conformational changes, increasing SOD1 β-sheet structures and consequently increasing neurotoxic SOD1 aggregation into the cells, therefore promoting disease progression (200). SOD2 is involved indirectly in increased Ca^2+^ accumulation by inhibiting PTEN activation. H_2_O_2_ produced by SOD2 induces oxidative inactivation of PTEN by conversion of the sulfhydrul groups to a disulfide causing more compact protein structure. The PTEN tumor suppressor inhibits PI3K-derived PIP2 catalysis to PIP3. SOD2 function then increases the cellular content of PIP2, which also functions as a mediator of GPCR signaling, stimulating the PLC-IP3/DAG signaling pathway and leading to increased Ca^2+^ synthesis.

Interestingly, increased Ca^2+^ has been shown to activate the PKC-ERK1/2 cascade, which increases *SOD2* expression, therefore suggesting a feedback loop in the regulation of SOD2 activation (62, 127, 128) (Fig. 8B). Ca^2+^ signaling was shown to regulate SOD3 expression as the GPCR-PLC-Ca^2+^ signaling pathway increases *SOD3* mRNA expression to stimulate thyroid cancer cell proliferation, as mentioned above (184) (Fig. 8B). The reciprocal action of ROS and Ca^2+^ is not limited to redox enzyme activation but plays a prominent role in cellular energy production by promoting ATP synthesis, the Krebs cycle, and oxidative phosphorylation in mitochondria. Ca^2+^ is even needed for neutrophil activation and in phagosome formation, suggesting a role in the host defense against invading microbes (112) and demonstrating the ample responsibility in the regulation of cellular functions.

### C. RTK and small GTPase signaling

The RTK family contains 58 members divided into 20 classes mediating the signaling response of growth factors, cytokines, and hormones into the intracellular signaling network. Ligand binding to the receptor induces structural changes, stimulating the tyrosine kinase phosphorylation activity of downstream signaling molecules or neighboring RTK (202). SOD3 has been shown to stimulate RTK phosphorylation by the increased production of H_2_O_2_ (190). The mechanism underlying SOD3-derived RTK phosphorylation in the absence of supplemental ligands may be related to the ability of H_2_O_2_ to affect PTPs, thereby allowing RTK activation (266). This is supported by data suggesting that SOD3 overexpression nonspecifically increases the phosphorylation of RTKs stimulating subsequent membrane-associated SRC family phosphorylation and small GTPases RAS, RAC, CDC42, and RHO (190, 197) (Fig. 8C).

The small GTPases are monomeric proteins that appear active when associated with GTP and inactive when they hydrolyze GTP to GDP. The activity of small GTPases is controlled by small GTPase regulatory proteins GEFs, GAPs, and GDIs. GEF stimulates GTP loading to small GTPase, thus activating it, whereas GAP catalyzes the hydrolysis of GTP to GDP, and GDI inhibits GDP dissociation from small GTPase and maintains small GTPase in the cytosolic compartment. Once GDI and GAP expression is downregulated, small GTPase associates in the GTP bound active form at the cell membrane by the action of GEF (26, 73). Interestingly, small GTPase activity is affected also by SOD1, especially by mutant SOD1, by direct interaction, as demonstrated in ALS models.

As described above, the interaction between small GTPase RAC1 and SOD1 is enhanced by mutations in the *SOD1* gene that maintains downstream RAC1 signaling, leading to increased inflammatory cytokine production and the risk of ALS development (120, 157) (Fig. 8C). In addition to being involved in cytokine and ROS production, as part of the NADPH oxidase complex, RAC is engaged in cell cycle progression, transformation, induction of actin polymerization, regulation of membrane ruffling, and adhesion of cells to the ECM or neighboring cells, thereby regulating movement, tissue morphogenesis, cancer initiation, and metastasis (229, 361).

### D. Oncogene signaling

The activation of individual oncogenes mediates redox signal transduction to downstream target molecules (Table 1). The SRC proto-oncogene has been demonstrated to activate NADPH oxidases NOX1-, NOX3-, NOX4-, and NOX5-induced O_2_^•−^ production in cancer models (87, 105, 107, 171) and to increase mutant SOD1 aggregate formation in ALS. Remarkably, SRC signal transduction can selectively activate only NOX1 and NOX3 without activating NOX2 or NOX4 in cases where the signaling is mediated by downstream TKS4 and TKS5 (107).

**Table 1.** Growth Promoters Affecting Reduction/Oxidation Gene Activation

*Growth promoter*	*Associated redox gene*	*References*
BRAF	SOD3	(33, 196)
cABL	NOX5	(86)
E1A	NOX4	(34)
EGF	SOD1	(157)
EGFR	NOX3, NOX4	(170, 172, 203)
FLT-ITD	NOX4	(154, 155)
IGF	SOD1	(157)
MCF7	SOD1	(272)
MCF-10A	SOD1	(272)
MDA-MB157	SOD1	(272)
MDA-MB-231	SOD1	(272)
PDGF	NOX1, NOX5	(153, 187)
PI3K	NADPH oxidase complex activation	(139)
PKA	NOX1	(71, 181, 388)
PKC	NOX1, NOX2	(107, 332, 388)
RAS	NOX1, NOX4, SOD2, SOD3	(5, 34, 39, 188, 189, 195, 196, 272)
RGS4	SOD3	(189)
SRC family	NOX1–5, SOD3	(45, 56, 104, 106, 170, 172, 322)

EGF, epidermal growth factor; FLT-ITD, FMS-like tyrosine kinase with internal tandem duplication; IGF, insulin growth factor; PDGF, platelet-derived growth factor; PI3K, phosphatidylinositol-4,5-bisphosphate 3-kinase; PKA, protein kinase A; PKC, protein kinase C; redox, reduction/oxidation; RGS4, regulator of G protein signaling 4; SOD, superoxide dismutase.

In primary cell transformation, SRC interacts with small GTPases by inducing persistent activation of RAC1 through tyrosine phosphorylation of RAC1 GEF VAV2 (105, 171, 173, 323). Furthermore, RAC1 has a crucial role in RAS oncogene-induced ROS production and activates oncogene-induced proliferative bursts, growth arrest, and escape from premature senescence, consequently causing immortalization and transformation of primary cells (263, 321, 322).

EGFR-SRC signaling-stimulated NADPH oxidase NOX4 enhances anoikis (cell detachment-induced apoptosis) resistance and survival of cancer cells, which are detached from the ECM but are still anchorage dependent and thereby sensitive to anoikis. Characteristically, cells that are released from stromal components lose the survival signaling provided by integrins that cause cell death initiation. However, cancer cells may gain resistance to anoikis, enabling the cells to intravasate, survive in vasculature, and extravasate into distant tissue sites (341, 344).

Involvement of SRC in redox signaling has also been investigated in neuronal degeneration models. A recent study utilizing induced pluripotent stem cells (iPSCs) derived from ALS patient with SOD1 mutations suggested that increased phosphorylation of SRC-ABL (Abelson murine leukemia viral *oncogene* homologue 1) causes the degeneration of motor neurons differentiated from iPSCs. Interestingly, the inhibition of SRC-ABL inhibited motor neuron degeneration by reducing SOD1 misfolding and restoring energy homeostasis (144).

In oncogene-driven mammary tumor models, such as the MCF-10A, MCF7, MDA-MB-231, and MDA-MB157 cell lines and (MMTV)-ErbB2, MMTV-Myc, and MMTV-Wnt transgenic mice, SOD1 is overexpressed in 60–100% of cases (273), suggesting that SOD1 activation is not linked to a specific oncogene and does not solely depend on oncogene activation. In contrast, SOD2 is downregulated by oncogenes, such as RAS, in the early phases of carcinogenesis (273), whereas RAS activation initially increases SOD3 production at low oncogene activation levels with consequent downregulation of SOD3 at high oncogene activation levels (33).

## XIII. Altered ROS Levels Are Associated with Progression of Tumorigenesis

In numerous cancers, aberrant ROS production regulates proliferation, survival, angiogenesis, and stroma remodeling. NADPH oxidase-derived sustained ROS production in the liver can contribute to chronic inflammation, which in certain cases may result in hepatocellular carcinoma development (89). ROS synthesis by NADPH oxidase NOX5 and SOD2 has been suggested to contribute to progression of gastroesophageal reflux disease, which characteristically exposes the esophageal tissue to acids causing increased intracellular Ca^2+^-ROCK2 signaling and upregulation of *NOX5* mRNA synthesis, to esophageal adenocarcinoma (139). NADPH oxidase NOX5-S-derived ROS then promote cell proliferation and survival (94), whereas SOD2, stimulated by NFκB and ZEB2, is involved in EMT of esophageal adenocarcinoma cells (175). The association of SOD2 with aggressive metastatic cancer phenotype is further supported by positive correlation of increased SOD2 expression with consequent H_2_O_2_-related *MMP9* and *VEGF* mRNA synthesis in bladder cancer (129).

In melanoma, ultraviolet (UV) radiation is the main external factor increasing ROS production, although the mechanism of melanoma molecular pathogenesis is heterogeneous. Skin exposure to UV results in a dose-dependent response in H_2_O_2_ production in melanocytes that directly corresponds to increased DNA damages. Noteworthy, somatic mutations, such as BRAF V600E, and activation of PI3K-AKT signaling pathway further increase ROS production, thus driving the progression of melanoma carcinogenesis (365). Increased expression of NADPH oxidase NOX4 has been shown to regulate cellular cycling, anchorage-independent growth, and *in vivo* tumorigenicity, thereby suggesting that oxidase is required for malignant transformation and increased tumorigenesis (389).

Other cancers that associate with NADPH oxidase NOX4 include prostate cancer, pancreatic cancer, and urothelial carcinoma. NOX4 is highly expressed in prostate cancer cell lines and in prostate cancer tissues compared with normal prostate cell lines or benign prostate tissues, respectively (243). NADPH oxidase NOX4 may further induce prostate tumor stroma fibroblast differentiation to myofibroblasts *via* activation of JNK and its downstream transcription factors, thereby coordinating TGFβ response in stroma remodeling (312). In pancreatic cancer models, H-RASV12 oncogene induces ROS production in RAC1 and NADPH oxidase NOX4-dependent manner (263).

ROS then promote SRC-mediated phosphorylation of protein kinase D1 at Tyr95, Tyr432, Tyr436, and Tyr502 with consequent activation of NFκB, increased expression of RTK EGFR, and its ligands EGF and TGFα. NFκB may also be involved in the activation of SOD2, thus increasing the mitochondrial ROS production causing mitochondrial dysfunction (80). In urothelial carcinoma, NOX4, which is detected already in precancerous and in early noninvasive urothelial carcinoma, has been shown to stimulate cell cycle progression and cancer cell growth (329). Therefore, the continuous reciprocal interaction of redox enzymes and cancer-related signal transduction pathways regulates the redox balance and cancer progression.

## XIV. ROS in Thyroid Cancer

### A. Redox-related signaling in thyroid tumorigenesis

Thyroid tumors are among the most thoroughly characterized neoplastic diseases consisting of both benign and malignant forms, hence forming a well-characterized platform for redox enzyme studies. In thyroid tissue, TSHR, representing GPCR, mediates downstream signaling *via* cAMP-PKA and PLC-Ca^2+^ pathways, which stimulate redox enzyme expression. In the pathway leading to thyroid hormone T_3_ and T_4_ synthesis, GPCR subunits Gαq/11 and Gβγ stimulate PLCβ, which recruits IP3 and DAG formation, eventually increasing Ca^2+^ mobilization and activating downstream signaling, leading to thyroid cell differentiation and thyroid hormone production (166). Aberrations in the availability of the thyroid-stimulating hormone (TSH) ligand or mutations in downstream cascades disrupt the delicate signaling network, resulting in various thyroid pathologies; thus, the constant stable level secretion of TSH is important for normal thyroid function. Decreased iodide uptake results in reduced T_3_ and T_4_ hormone synthesis (hypothyroidism), which is compensated by increased TSH production.

Increased TSH levels activate downstream growth signal transduction, causing thyroid enlargement known as goiter, the most common thyroid disorder characterized by hyperplasia and hypertrophy. Although goiter can be associated with autonomous adenoma, histologically and functionally, it differs from thyroid adenomas (380).

Thyroid tumors originate from follicular and parafollicular C cells by genetic and epigenetic alterations, with consequent uncontrolled activation of signaling pathways maintaining thyroid cancer progression. Until now, the observed mutated genes include *BRAF*, *RAS*, phosphatidylinositol-4,5-bisphosphate 3-kinase catalytic subunit α (*PI3Kα*), *PTEN*, *TP53*, *β-CATENIN*, anaplastic lymphoma kinase (*ALK*), isocitrate dehydrogenase 1 (*IDH1*), rearrangement of rearranged during transfection (*RET*)-*PTC*, and paired box 8 (*PAX8*)-peroxisome proliferator-activated receptor γ (*PPARγ*) resulting in aberrantly activated signal transduction pathways downstream of mutated genes. *RAS* and *BRAFV600E* mutations constitutively activate the Ras Association Domain Family Member 1 (RASSF1)—mammalian STE20-like protein kinase 1 (MST1) —FOXO3 signaling pathway, and MEK1/2-ERK1/2 pathway (36, 43, 383).

Although the initiation of thyroid cancer is well described in the literature, the progression toward more aggressive metastatic phenotypes is more complex, affecting simultaneously many signaling pathways. Incurable ATC pathogenesis is associated with the activation of platelet-derived growth factor receptor (PDGFR), VEGFR, EGFR, hepatocyte growth factor receptor (HGFR, c-MET), and proto-oncogene c-KIT (CD117), a type III receptor tyrosine kinase. The activation of cell membrane signaling molecules then causes persistent phosphorylation of downstream cell survival, angiogenesis, antiapoptotic, and cell proliferation, supporting PI3K-AKT and MAPK signaling (364, 383). The PI3K-AKT pathway is activated by a mutation in *PI3Kα* encoding the catalytic p110α subunit or in the *PTEN* phosphatase gene that functions as a tumor suppressor regulating PI3K activity.

The PI3K-AKT pathway plays a central role in the development and progression of follicular thyroid carcinoma, which is characterized by vascular and capsular invasiveness. In capsular invasion, thyroid tumor cells invade into the peritumoral connective tissue serving as a marker of benign to malignant progression (104, 383). Mutations affecting the pathway are less common in follicular thyroid adenoma than in follicular thyroid carcinoma, and therefore, mutations affecting PI3K activity may promote the conversion of adenoma cells to carcinoma cells. The role of NADPH NOX enzymes is poorly characterized in the development of follicular thyroid cancer, although PI3K-AKT pathway activation is required for the phosphorylation of p47^phox^ initiation of NADPH NOX assembly, suggesting a role for ROS in the benign to malignant transformation, invasion of cancer cells, and metastatic cancer niche formation (45, 57, 122, 253, 334, 383). As mentioned previously in this review, SOD2 overexpression induces cell migration by activating p130Cas (BCAR1), consequently causing the inactivation of PTEN (127). Interestingly, p130Cas has been shown to interact with thyroid hormone receptor interactor 6 (TRIP6), causing increased MMP expression and the initiation of the invasion program (390). Therefore, signal transduction pathways driving thyroid carcinogenesis may simultaneously upregulate ROS-producing enzymes that participate in cancer progression.

In thyroid tumors, similar to most solid tumors, the paracrine secretion from the stroma is involved in the regulation of epithelial cancer cell growth and migration. Stromal cells secrete a high number of cytokines, growth factors, and ROS that contribute to fibroblast differentiation to activated myofibroblasts, a cornerstone of fibrotic stroma development. An interesting characteristic of PTC stroma is the coexistence of dense desmoplastic stromal regions and highly vascularized inflammatory cell-rich regions (275).

Inflammation is characteristic of highly vascularized stroma, whereas fibrosis and poorly vascularized connective tissue-rich desmoplasia commonly represent the last stromal developmental stages. Increased inflammatory cell migration is characteristic of developing tumors and, in some cases, may even be a cause of malignant transformation. Inflammatory cells secrete a high number of cytokines, growth factors, and ROS, which support epithelial cancer cell growth and tumor microenvironment maturation by regulating growth, migration, and differentiation (35, 40).

Although chronic inflammation is associated with premalignant lesion development, the inflammation itself cannot be considered tumorigenic. Instead, the recruitment of inflammatory cells, such as cytotoxic T cells and natural killer (NK) cells, to developing cancer, may represent a failed mechanism of an attempt of the tissue to defend itself against tumorigenesis. Indeed, a recent development of immunotherapy by disrupting the connection of programmed death 1 (PD-1), expressed by T cells, and the ligand programmed death ligand 1 (PD-L1) and programmed death ligand 2 (PD-L2), expressed by cancer cells, disrupts a crucially important signaling cascade meant to prevent tumor escape from immune destruction (79). In the later phases of carcinogenesis, cancer cells have been shown to educate inflammatory cells toward the protumorigenic mode, indicating the ability of cancer cells to adapt and utilize host tissue defense functions (46).

As part of the last developmental phase of granulation, highly vascularized stroma is gradually replaced by poorly vascularized fibrotic tissue largely composed of collagen secreted by myofibroblasts. NAPDH NOX4-derived ROS have been shown to play a major role in the differentiation of fibroblasts into myofibroblasts, which have been shown to originate from fibroblasts or directly from MSCs (206, 275). Myofibroblasts express MMPs that can remodel the desmoplastic stroma, thus facilitating the local migration of cancer cells and metastasis to distant organs. NADPH NOX1 and especially SOD2 have been shown to stimulate MMP expression and to interrupt focal adhesions as part of the migration/invasion process in cancer progression. In thyroid cancers, fibrotic reaction and desmoplasia correlate with lymph node metastasis and are, therefore, used as a clinical diagnostic marker. Interestingly, desmoplasia develops in the relatively early phase of thyroid tumorigenesis, being present even in 80% of medullary thyroid cancers and correlating with increased ROS production and increased redox gene expression in stromal cells. Importantly, primary tumor stroma and metastasis site microenvironment are under constant development as the stroma responds to local epithelial cancer cell needs—nurturing the growth and migration requirements. Thus, the tumor stroma contains different developmental stages from early-stage vascular permeability and extravasation of fibrinogen to late-phase increased myofibroblast collagen production (177, 178, 317).

### B. *NOX1–5* and *SOD1–3* expression in thyroid cancer

**FIG. 9.**
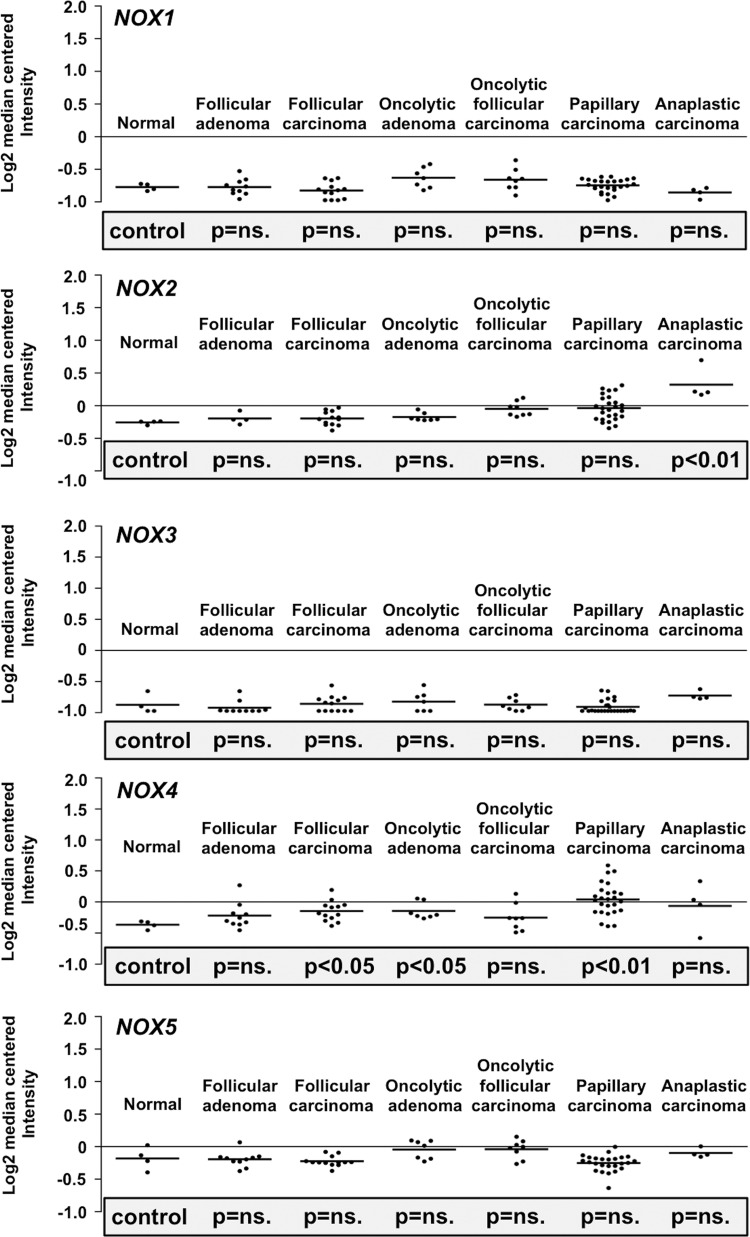

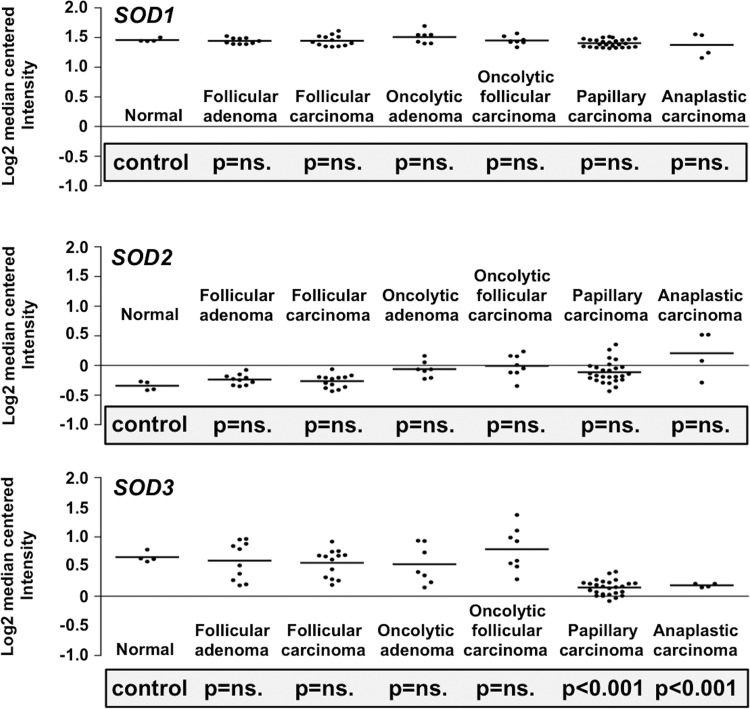
**NOX1-5 and SOD1-3 gene expression inn thyroid cancers extracted from Oncomine Giordano database.** Gene expression values were collected individually from the database in each thyroid cancer and presented as Log2 mediancentered intensity expression. The *p*-values (*p* < 0.05, *p* < 0.01, *p* < 0.001) were determined by two-tail independent samples t-tests comparing to normal thyroid expression level.

Interestingly, ROS, especially H_2_O_2_, are produced in the thyroid at levels that are normally toxic to cells. Dual oxidases (227) and NOX complexes have been reported to be the main sources of H_2_O_2_ in the thyroid, although the relative expression of *NOX1–5* is markedly lower than that of *SOD1–3* enzymes. The expression levels of *NOX1–5* and *SOD1–3* genes in the normal thyroid, follicular adenoma, follicular carcinoma, oncolytic adenoma, oncolytic follicular carcinoma, papillary carcinoma, and anaplastic thyroid carcinoma extracted from the Oncomine Giordano database (109) (Fig. 9) demonstrate the relatively low expression of *NOX1–5*.

The average *NOX1* Log2 median-centered intensity-normalized expression in the normal thyroid is −0.77 and remains at that level in thyroid tumorigenesis showing −0.75 and −0.85 Log2 median-centered intensity-normalized expression in papillary thyroid and anaplastic thyroid cancers, respectively. The normal thyroid average *NOX2* Log2 median-centered intensity-normalized expression (−0.26) is significantly (*p* < 0.01) increased in anaplastic thyroid cancer that characteristically has massive inflammatory cell infiltration, explaining the presence of hematopoietic cell NOX2. The average *NOX3* Log2 median-centered intensity-normalized expression is extremely low in the thyroid, reflecting its unique role in the regulation of the differentiation of progenitor cells (269).

The association of *NOX3* expression with primitive cells is further corroborated by observations suggesting modestly increased mRNA in undifferentiated ovarian teratocarcinoma and adenocarcinoma cancer cells (50). However, the corresponding Log2 median-centered intensity expression for *NOX3* in the normal thyroid is −0.87 and remains at that level even in undifferentiated anaplastic thyroid cancer. NOX4, which has a prominent role in cancer and tumor stroma progression, shows an increased average Log2 median-centered intensity in thyroid tumorigenesis. The expression of *NOX4*, which in the normal thyroid is −0.37 similar to the level of *NOX2*, shows a moderate but significant increase in follicular carcinoma (−0.22; *p* < 0.05), oncolytic adenoma (−0.15; *p* < 0.05), and especially in papillary thyroid carcinoma (0.04; *p* < 0.01), characterized by desmosplastic stroma development (Fig. 9).

Interestingly, the database has suggested relatively high *SOD1* and *SOD3* gene expression in general in normal thyroid tissue and thyroid tumors. The average expression for *SOD1* shown as Log2 median-centered intensity in the normal thyroid is 1.46 and remains at the same level in thyroid tumors. The average *SOD3* Log2 median-centered intensity in the normal thyroid is 0.66, which is then decreased gradually, showing the lowest values in PTC (0.15; *p* < 0.001) and in anaplastic thyroid cancer (0.18; *p* < 0.001). The average Log2 median-centered intensity-normalized *SOD2* expression in the normal thyroid is markedly lower, −0.34, than that of *SOD1* and *SOD3* but different from these two because *SOD2* expression gradually increases, correlating with the malignancy of thyroid cancer. There is a moderate increase in follicular adenoma and in the corresponding adenocarcinoma, as well as in oncolytic adenoma and in the corresponding oncolytic follicular carcinoma. The highest increase in *SOD2* expression (0.21) is observed in undifferentiated anaplastic carcinoma, a highly aggressive metastatic tumor. The markedly increased expression of *SOD2* in metastatic anaplastic thyroid cancer may be causally related to cancer-related modifications of tumor cell metabolism. The high overall expression levels of *SOD1* and *SOD3* may instead indicate significant cytoplasmic and extracellular H_2_O_2_ production (Fig. 9).

### C. Percentage change in redox gene expression in PTC

**FIG. 10.**
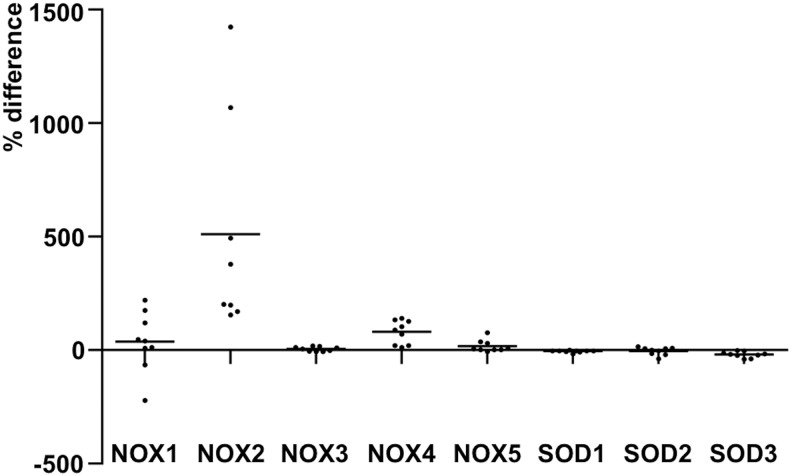
***NOX1–5*** **and** ***SOD1–3*** **gene expression in papillary thyroid cancer extracted from Oncomine He database.** Gene expression values in papillary thyroid cancer were compared with the normal thyroid expression values of the same patients. The results are shown as percentage change of the Log2 median-centered intensity expression level.

In general, the gene expression levels vary significantly among individual persons, thus requiring a high number of patients in each study group or alternatively a control sample from the same patient to make definitive conclusions. To confirm the data extracted from Giordano's thyroid database, we normalized mRNA expression in PTC against normal tissue isolated from the same patient using Oncomine's He's database (124). The Log2 median-centered intensity data presented in Figure 10 are supported by the analysis of the percentage increase of *NOX1–5* and *SOD1–3* in PTC compared with normal thyroid expression levels. In He's database, there were nine patients who had both normal and papillary thyroid tissues available for the expression analysis. The percentage of *NOX1–5* expression in PTC compared with normal tissue expression levels suggests a 37.5% (standard devation [SD] 131.4) increased expression for *NOX1* and a noticeable 532.5% (SD 513.7) increase for *NOX2*, indicating PTC inflammatory cell infiltration. The average expression of *NOX3* was at the level of normal thyroid (5.2% increase [SD 9.9]), whereas *NOX4* showed a marked 80% (SD 51.4) average increase from the basic expression levels. *NOX5* expression demonstrated a moderate 17.6% (SD 27.4) increase in PTC tissue (Fig. 10). The differences strengthen the observation suggesting high variation among individuals, obligating the use of many patients in each study to convincingly conclude the expression data and, more importantly, redox enzyme-derived effects on downstream signal transduction pathways.

*SOD1* expression was decreased in eight of nine patients, *SOD2* expression was decreased in four of nine patients, and *SOD3* expression was decreased in all nine patients. Therefore, the final conclusions in estimating redox gene expression in patient samples should be associated with the consistency of gene expression. The comparison of the average *SOD1*, *SOD2*, and *SOD3* mRNA synthesis in normal and PTC showed 4.6% (SD 4.9), 3.5% (SD 17.1), and 18.6% (SD 13.1) decreased expression in cancer, respectively (Fig. 10). Although *SOD1* and *SOD2* expression levels in He's database are opposite compared with those in the Giordano's database, the differences can be considered minor and might fit between markedly high SDs. Decreased *SOD3* is detected in both databases, confirming the earlier observations (184) and suggesting SOD3 as a differentiation marker in thyroid tumorigenesis.

Thus, the Oncomine Giordano and He database results showing thyroid cancer-related redox gene expression suggest that *NOX1*, *NOX3*, and *NOX5* mRNA levels do not markedly change in tumorigenesis, whereas *NOX2* and *NOX4* expression showed increased levels correlating with the degree of transformation. *NOX2*, which is associated with inflammatory cells, is mostly increased in oncolytic follicular carcinoma, papillary thyroid carcinoma, and, especially, in anaplastic thyroid carcinoma. Of note, anaplastic thyroid carcinoma is marked by the constitutively activated PI3K-AKT pathway, which is needed for p47^phos^ NOX2 subunit phosphorylation and NADPH NOX2 complex assembly (383). *NOX4* expression is moderately increased in all thyroid tumors, which may be related to the role of the tumor stroma paracrine effect. Indeed, *NOX4*-produced ROS have been reported to induce fibroblast differentiation to myofibroblasts, thus indicating a growth supportive role for *NOX4* in thyroid carcinogenesis.

## XV. ROS in Colon Cancer

### A. Progression of colon cancer

Colon cancer, similar to thyroid cancer, is a well-characterized disease, which often progresses from benign adenoma to malignant adenocarcinoma and gains oncogene mutations influencing signal transduction, thereby altering redox gene expression. Colon tumorigenesis is caused by genotoxic stress-derived protumorigenic DNA mutations, activation of oncogenes, and inactivation of tumor suppressor genes in which ROS have a contributory function (37, 116, 313). Inherited conditions, such as hereditary nonpolyposis colorectal cancer (HNPCC) and familial adenomatous polyposis (FAP), markedly increase the sensitivity to develop malignant carcinoma. Colon tumorigenesis can be divided into three different phases based on the abnormalities observed in colon epithelial cells: initiation, promotion, and progression. In the initiation phase, epithelial cells accumulate DNA damage spontaneously or by the action of protumorigenic factors that then drive the development of aberrant cystic foci, increasing colon cancer incidence. Characteristically, these early events include inactivating mutations in the adenomatous polyposis coli (*APC*) tumor suppressor gene that controls growth-promoting WNT signaling by forming the β-catenin destruction complex together with axis inhibition protein (AXIN), CK1, glycogen synthase kinase 3β (GSK3β), causing cytoplasmic degradation of β-catenin (251). Mutations in *APC* result in the disrupted function of the β-catenin destruction complex, increased accumulation of β-catenin in the cytoplasm, and eventually increased β-catenin translocation into the nucleus. In the nucleus, β-catenin interacts with T cell factor/lymphoid enhancer binding factor (TCF/LEF), inducing the expression of WNT target genes, such as *CYCLIN D1* and *cMYC* (42). Recently, the importance of changes in colon microbiota has been suggested to increase cancer risk by producing bacterial toxins, such as colibactin. ROS O_2_^•−^ synthesized by *Escherichia coli* and *Enterococcus faecalis* is spontaneously dismutated to H_2_O_2_ in the colon environment increasing oxidative stress and consequent DNA base modifications, DNA-protein crosslinks, and DNA single- or double-strand breaks. Colibactin secreted by *E. coli* can cause transient DNA damage, incomplete DNA repair, anaphase bridges, and chromosomal aberrations in mammalian cells even after a short exposure (66).

In the promotion phase, intestinal cells form benign, early, intermediate, and late adenomas. A recent work demonstrated the recruitment of ERK5 signaling in colon carcinoma HCT116 cells to compensate for decreased ERK1/2 kinase phosphorylation allowing the progression of cell proliferation and tumor formation. Although the mitogenic ERK1/2 pathway is the main proliferative signaling route, the study suggested incomplete intestinal epithelial cell maturation and migration along the crypt/villus axis in the absence of phosphorylated ERK1/2, indicating the importance of ERK1/2 for normal colon function (71).

Benign colon tumors, such as polyps, can be either mushroom-shaped protrusions inside the large intestine or flat growths in the intestinal wall. Most of the polyps are harmless, but the risk of protumorigenic mutations increases correlating with the size of the polyp. Diminutive (1–5 mm in size) and small (6–9 mm in size) polyps are virtually always benign, whereas large polyps over 10 mm diameter have an increased tendency to accumulate carcinogenic characteristics. Although polyps are common, the frequency to have carcinoma progression from polyps is low, ∼1% of cases. However, in the case of HNPCC and FAP, the risk of malignant transformation is markedly higher, approximately 50–60% in HNPPC patients and over 90% in FAP patients, correlating with the age of the person (115).

In the third progression phase, colon cells in the polyps that have gained a carcinogenic phenotype initiate hyperproliferation, initiating colon cancer progression. Although the nonmetastasized adenocarcinoma can be surgically removed, occasionally it develops into metastatic colon cancer. The mechanism underlying the progression of primary colon adenocarcinoma to metastatic cancer is not completely understood; thus, the disease remains highly lethal with a median survival of 20–36 months. Metastatic cancer progression has been shown to commit mutations affecting proto-oncogenes, especially those of *KRAS*, *NRAS*, and *BRAF* that promote cellular migration and invasion, causing characteristic metastasis to the liver, lung, bone, and brain. The overall survival is correlated with the mutations observed—for example, *BRAFV600E* mutation predicts nodal and peritoneal metastasis and a poor prognosis (355). The accumulation of genetic lesions in tumor suppressor gene *TP53* and the switch of the tumor suppressor TGFβR-SMAD pathway into a tumor promoter by mutation in SMAD4 are further drivers of tumor progression, indicating the complex nature of the disease (152). Other noteworthy mechanisms promoting cancer cell migration include EGFR downstream activator son of sevenless (SOS) and growth factor receptor-bound protein 2 (GRB2) that increases PI3K-AKT signaling (152).

### B. WNT signaling in the normal colon and in colon cancer development

Int/Wingless (WNT) signal transduction is crucial in the maintenance of colon stem cells and progenitor cells, which then differentiate into colon epithelial cells. In general, WNT ligand signals through a canonical pathway regulating β-catenin entry into the nucleus and through the noncanonical RAS-ERK1/2 and WNT/Ca^2+^ pathways stimulating Ca^2+^-related signaling molecules CAMKII, PLC-PKC, and calcineurin. Activation of canonical signaling contributes to fibroblast activation and fibrosis development, although it has been demonstrated to cause the development and progression of cancer in various model systems (42).

The canonical signal transduction is initiated by WNT ligand binding to the transmembrane frizzled (FZD) receptor, which destabilizes the β-catenin destruction complex allowing β-catenin accumulation into the cytoplasm and consequent entry into the nucleus. Downregulation of casein kinase 2 (CK2), which is a positive regulator of WNT signaling and is required for WNT ligand secretion from WNT-producing cells (70), induces PI3K-AKT phosphorylation with consequent phosphorylation and inactivation of FOXO3a in colon cancer cells. According to previous publications, phosphorylated FOXO3a is translocated from the nucleus into the cytoplasm, inactivating the mRNA synthesis of the target genes *SOD1*, *SOD2*, and *catalase* but also increasing *mir21* expression, which targets *SOD3* mRNA 3′UTR, resulting in degradation of mRNA (184, 279, 363, 396). Therefore, downregulation of CK2 and consequent impaired WNT signaling may markedly influence the accumulation of O_2_^•−^ into cells, likely resulting in colon cancer cell proliferation, survival, and senescence.

The noncanonical β-catenin-independent signal transduction pathway recruits small GTPase RAC1 and RHOA regulating cytoskeletal remodeling and cell motility *via* ROCK and JNK activation, whereas the noncanonical WNT/Ca^2+^ pathway controls cell migration and is involved in inflammation and in cancer promotion. Noncanonical WNT/Ca^2+^-dependent signal transduction can activate PKA/CREB and p38/activating transcription factor 2 (ATF2) signaling molecules or, more interestingly, the PI3K-AKT pathway, which is involved in NADPH-NOX p47^phox^ activation (180). This is supported by observations demonstrating NOX1 participation in the recovery from colon inflammation injury, increasing cell proliferation, migration, terminal differentiation, and antiapoptotic activity (159, 164).

### C. *NOX1–5* and *SOD1–3* gene expression in colon tumorigenesis

**FIG. 11.**
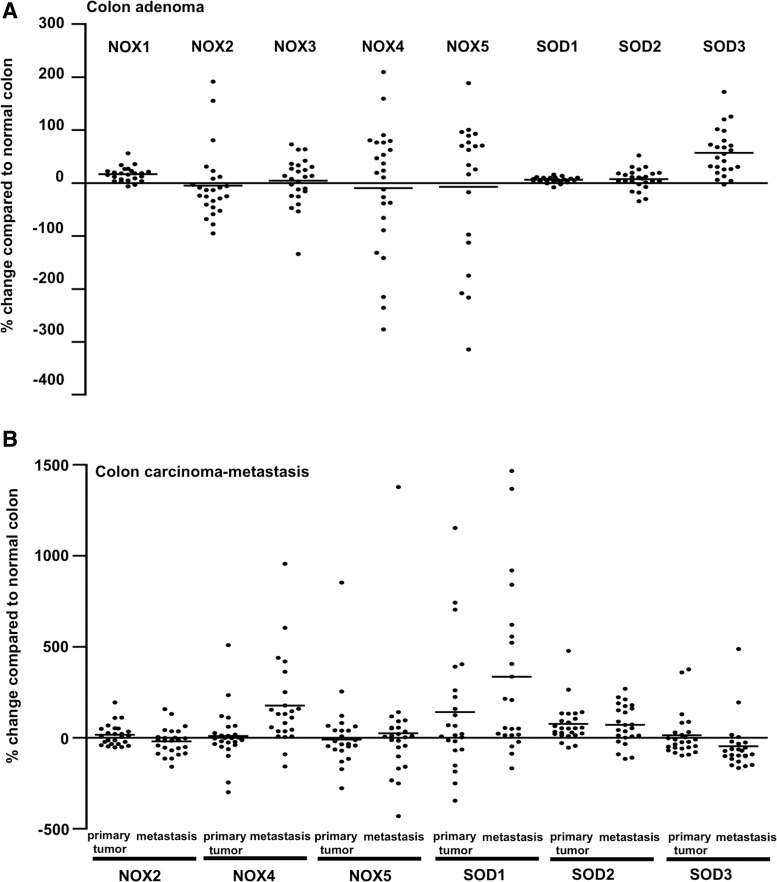
***NOX1–5*** **and** ***SOD1–3*** **gene expression in colon tumors extracted from Oncomine Sabates-Bellver and Ki databases. (A)** *NOX1–5* and *SOD1–3* expression in benign colon adenoma normalized against the normal colon tissue of the same patient. The data suggest high differences between individuals in redox gene expression. **(B)** *NOX1–5 and SOD1–3* expression in primary colon adenocarcinoma tumor and in metastasis extracted from Ki colon database. Database analysis suggests increased *SOD1*, *NOX4*, and *NOX5* expression in metastasis, stable *SOD2* expression, and decreased *SOD3* and *NOX2* expression in metastasis.

We utilized the Oncomine Sabates-Bellver database (305) to define redox gene expression in benign colon adenoma clinical samples (Fig. 11A). To diminish the variation among individuals, we calculated the percentage difference in gene expression in adenoma tissue compared with normal tissue expression level in the same patient. Interestingly, the average *NOX* mRNA expression showed a high degree of variation among individuals. The average *NOX1* and *NOX2* expressions in a benign colon tumor suggested a 16.9% (SD 13.66) increase and a 4.8% decrease (SD 65.3), respectively. However, the remarkable high SDs invalidate all definitive conclusions of *NOX2* expression levels in colon tumorigenesis. Similar outcome revealed *NOX3*, *NOX4*, and *NOX5* mRNA expression levels with a 4.3% increase, a 9.4% decrease, and a 48.7% decrease in expression, respectively. The SDs then nullify the average expression data showing SD 44.2 for *NOX3*, SD 119.5 for *NOX4*, and SD 185.7 for *NOX5* expression in different patients.

As shown in Figure 11A, *SOD1* mRNA expression in colon adenoma is increased by 6.2% (SD 5.2), indicating relatively stable *SOD1* expression in the early phase of tumorigenesis. *SOD2* expression is increased by 7.6% (SD 19.2) and *SOD3* by 57.3% (SD 43.5), suggesting a correlation between *SOD3* expression and benign tumorigenesis. Similarly, in adenoma development-related *NOX1–5* and *SOD1–3* gene expression, the progression of primary colon adenocarcinoma to metastatic cancer shows large variation in redox gene expression in individual patients compared to the normal colon tissue values.

The Ki (167) database reporting gene expression in the normal colon, adenocarcinoma, and metastatic colon cancer (Fig. 11B) does not show any values for *NOX1* or *NOX3* expression, but *NOX2* expression suggests relatively stable mRNA levels, showing, on average, a 16.9% (SD 60.0) increase in the primary tumor and a 19.0% (SD 74.0) decrease in metastasis (Fig. 11B). *NOX4* expression is approximately at the levels of normal tissue, suggesting a mild 11.6% (SD 146.8) average increase in primary colon carcinoma, whereas there is an average increase of 178.3% (SD 244.7) in metastasis. *NOX5* mRNA expression suggests minimal average changes showing an 8.4% (SD 256.9) decrease in primary cancer and a 26.3% (SD 317.4) increase in metastasis (Fig. 11B).

The database suggests increased overall mRNA synthesis for *SOD1* and *SOD2* in adenocarcinoma (142% and 77.1%, respectively), although the variations between patients are high as observed in adenoma development: SD 347.4 for *SOD1* and SD 108.1 for *SOD2*. The metastatic disease shows a 515% increase in *SOD1* expression and 73.2% increase in *SOD2* expression; however, again, the SDs, SD 972.6 for *SOD1* and SD 108.1 for *SOD2*, nullify the individual values.

Based on the Oncomine data, the average *SOD3* expression level is mildly increased by 14.8% (SD 121.0) in primary colon carcinoma tumor and moderately decreased by 46% (SD 136.5). Again, the variation among individuals nullifies the general conclusions of the expression levels; therefore, the effect of SOD3 in tumor progression cannot be generalized using patient expression data alone. However, when evaluating the patient data, it is important to note that several studies have suggested a role for SOD2 in the promotion of cancer cell migration and invasion (126, 165, 207). The extremely high SDs could causally indicate differences in the signal transduction pathway activation status in patients. Hence, any conclusions for redox gene expression-related data in the colon environment require accurate analysis of the tumor developmental stage, signal transduction pathways, possible effect of inflammation and microbiota, and an adequate number of patients. Therefore, expression data alone from clinical patients do not comprehensively predict the role of the enzyme in cancer progression. This is further supported by recent data showing increased carcinogenesis in cells with restored SOD3 expression. Virus-mediated gene transfer of *SOD3* to restore the expression of the enzyme is correlated with increased primary tumor growth and metastasis levels (368).

## XVI. ROS in Breast Cancer

### A. ROS-related characteristics of breast cancer

Breast cancer is frequently classified into four molecular subtypes: (i) luminal A subtype, which has high estrogen and progesterone receptor expression but low human epidermal growth factor receptor 2 (HER2) expression; (ii) luminal B subtype, which has lower estrogen and progesterone receptor levels and variable HER2 expression; (iii) basal-like triple negative subtype, which has negative estrogen receptor, progesterone receptor, and HER2 expression; and (iv) HER2 subtype, which has high HER2 expression but generally does not express estrogen and progesterone receptors (9). Breast cancer risk factors are frequently associated with oxidative stress-linked phenomena, such as aging, obesity, smoking, or alcohol consumption, and the pedigree of patients, which is the basis for the hereditary breast/ovarian syndrome that can be caused by mutations in *BREAST CANCER 1* (*BRCA1*), *BRCA2*, and *TP53* tumor suppressor genes (215, 238). Breast tumor expansion causes glucose deprivation, hypoxia, and oxidative stress due to lack of blood vessels. Elevated ROS production augments IL-8 and VEGF synthesis in breast cancer cells and enhances MMP secretion, supporting new blood vessel growth and cancer cell migration in the tumor microenvironment and consequently increasing the risk of breast cancer cell intravasation and metastasis (32, 83).

Tumor-associated macrophages are frequently observed in breast tumors and contribute to increased ROS production, directly or indirectly, by secreting ROS production-augmenting cytokines, thereby further increasing cancer cell migration, vascularization, and even relapse after chemotherapy treatment (143). Interestingly, a recent study identified a subpopulation of macrophages expressing mannose receptor C-type lectin (MRC1), angiopoietin receptor TIE2, VEGF-A, and CXCR4 in densely vascularized regions of breast tumors following chemotherapy; this finding suggests a potential role of macrophages in disease relapse (143).

As reported in other cancers, ROS are powerful DNA damaging agents that cause strand breaks, chromosomal rearrangements, and individual DNA base modification. In breast cancer, germ line mutations in *BRCA1* and *BRCA2* are examples of genomic instability that increases tumor susceptibility (257).

Previous studies have demonstrated upregulation of the expression of the NADPH oxidases NOX1, NOX4, and NOX5 in breast cancer, suggesting mitochondrial H_2_O_2_ production by NADPH oxidase NOX4, which in turn protects cancer cells against etoposide cytotoxin-induced cell death, increases invasion, anchorage-independent growth, and EMT (28, 113). Other studies have suggested that NADPH oxidase NOX4 promotes carcinogenesis and protects breast cancer cells from anoikis. Mechanistically, TGFβ secreted into the tumor environment causes phosphorylation of the SMAD2/3, SMAD complex and JNK activation, which in turn increases NADPH oxidase NOX4 synthesis. Alternatively, AKT1 phosphorylation may increase SP1 transcription factor synthesis and subsequent NADPH oxidase NOX4 production, which is paralleled with increased F-actin polymerization, invapodia formation, and increased invasion (28, 55, 351, 370).

In conjunction with the NAPDH oxidase NOX4, SOD2 contributes to breast cancer progression at late stage III by promoting AMPK phosphorylation at Thr172. AMPK then directs cellular oxidative respiration toward glycolysis, thus improving cell viability and inhibiting apoptosis (121).

### B. *NOX1–5* and *SOD1–3* gene expression in breast tumorigenesis

**FIG. 12.**
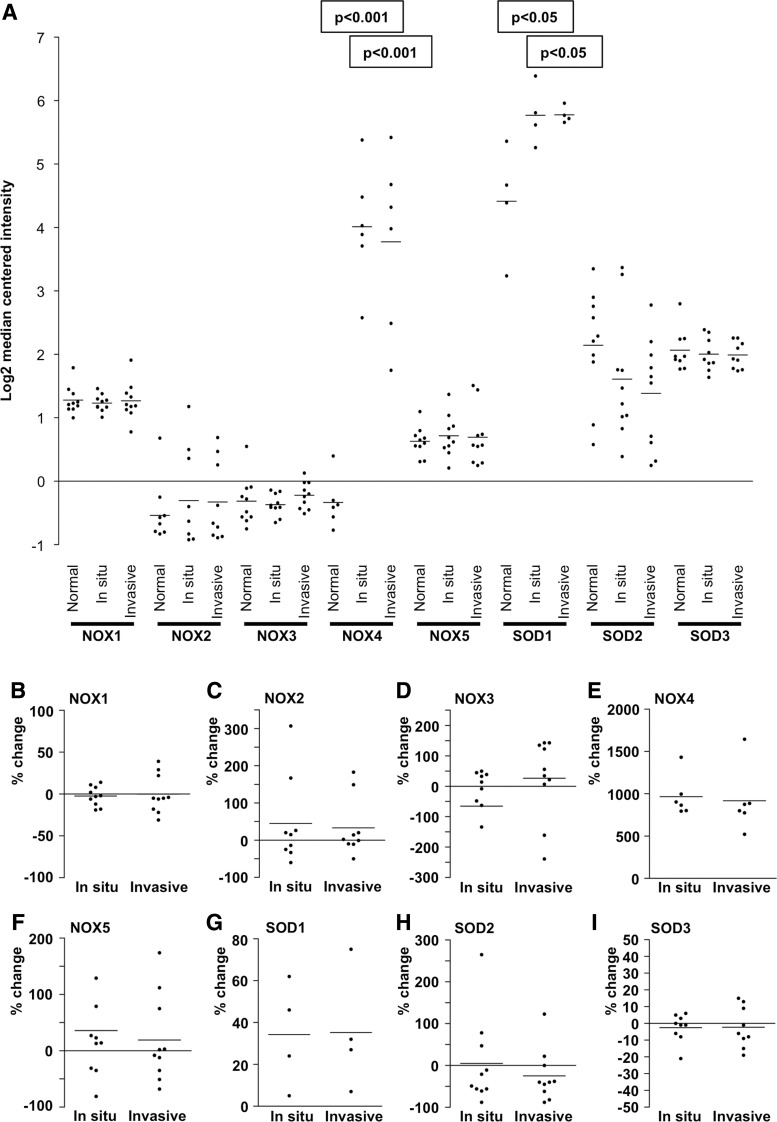
***NOX1–5*** **and** ***SOD1–3*** **gene expression in breast tumors extracted from the Oncomine Ma database. (A)** *NOX1–5* and *SOD1–3* expression in normal breast, in ductal breast carcinoma *in situ*, and in invasive ductal breast carcinoma of the same patient. NOX4 expression is significantly (*p* < 0.001) increased in both *in situ* and invasive ductal breast carcinomas. **(B–I)** Percentage change in *NOX1–5* and *SOD1–3* expression in ductal breast carcinoma *in situ* and in invasive ductal breast carcinoma as normalized against the normal breast tissue of the same patient. *p*-Values (*p* < 0.05, *p* < 0.01, *p* < 0.001) were determined by two-tail independent sample *t*-tests.

We extracted redox gene expression in patients with normal breast, *in situ* ductal breast carcinoma, and invasive ductal breast carcinoma from the Oncomine Ma database (210) (Fig. 12A). For the analysis, we included only patients from whom all three tissue types were isolated for the microarray. The analysis indicates relatively stable *NOX1*, *NOX2*, *NOX3*, and *NOX5* production, suggesting no significant differences at the expression levels between normal and tumor tissues, whereas *NOX4* expression is strongly upregulated in both *in situ* and invasive breast carcinoma. *SOD1* has high overall expression in breast tissue, and its expression is further increased in tumor tissues, whereas *SOD2* and *SOD3* are expressed at approximately levels twofold lower than *SOD1* levels.

Although *SOD2* expression is statistically at the same level in normal and pathological tissue, the data suggest a decreasing trend in the expression correlating to progression of tumorigenesis. We then calculated the percentage change in the redox gene expression levels in tumors compared to normal breast tissue from the same patient (Fig. 12B–I). Similar to that in thyroid and colon cancers, the individual differences nullified the statistical significance, thus corroborating the notion that redox gene expression alone may not predict carcinogenic progression.

## XVII. ROS in Lung Cancer

### A. ROS-related characteristics of lung cancer

Nonsmall-cell lung cancer (NSCLC) and small-cell lung cancer (SCLC) are the primary lung cancer types. NSCLC, which represents 75–80% of the cases and includes adenocarcinoma, squamous cell carcinoma, and large-cell carcinoma, originates from activating mutations in *EGFR*, *ALK*, *ROS 1 proto-oncogene receptor tyrosine kinase*, and *BRAF* genes. Characteristically, NSCLC may develop resistance to therapy by driver mutations in *EGFR*, *BIM*, *KRAS*, *MET*, and *HER2* that alter downstream signaling, allowing the tumors to bypass the upstream tyrosine kinase-targeted therapy (304). SCLC, which accounts for ∼15% of lung cancers, is a highly aggressive and highly vascularized metastatic form of cancer characterized by mutations in *TP53* and the transcriptional corepressor *RB1*, disruption of several signaling networks, and overexpression of MYC, MYCL, and MYCN transcription factors. Interestingly, small dense core granules identified as neuroendocrine cells in the tumors secrete adrenocorticotropic hormone, bombesin-like gastrin releasing peptides, and regulatory neuropeptides, thereby making SCLC a neuroendocrine lung tumor (41, 100, 191).

Although the oncogene mutations identified in lung cancers affect several signaling pathways that regulate redox enzyme expression, the role of ROS in lung carcinogenesis is not completely characterized. Studies performed in lung cancer models have resulted in interesting novel observations, such as NADPH oxidase NOX1-coordinated inhibition of p53 acetylation at Lys382 that affects p53 proapoptotic function; increased NOX2 expression in NSCLC patients, which correlates with decreased survival (293); and a novel role for the NADPH oxidase NOX3 in the regulation of redox balance in NSCLC causing a selective degradation of mutant EGFR (204). As reported previously, upregulation of the NADPH oxidase NOX4 contributes to myofibroblast activation and has a statistically significant correlation to patient survival (119). In addition, a recent study identified SOD1 as a tumor promoter in NSCLC, demonstrating that SOD1 inhibition with ATN-224 induces cellular death (110, 158, 274). Therefore, NADPH oxidases 1–5 and SOD1–3 potentially have prominent roles in lung carcinogenesis.

### B. *NOX1–5* and *SOD1–3* gene expression in lung tumorigenesis

**FIG. 13.**
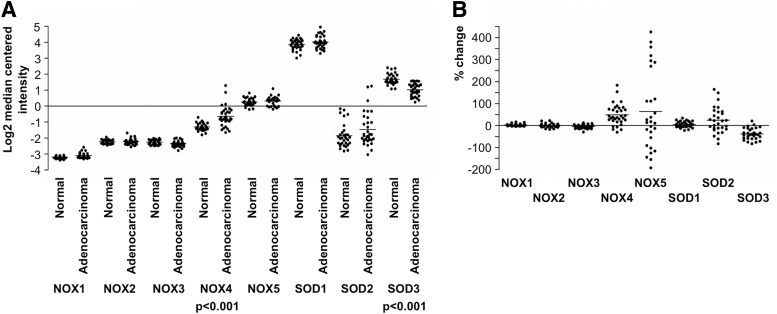
***NOX1–5*** **and** ***SOD1–3*** **gene expression in lung tumors extracted from the Oncomine Landi database. (A)** *NOX1–5* and *SOD1–3* expression in normal lung and in lung adenocarcinoma of the same patient. **(B)** Percentage change of *NOX1–5* and *SOD1–3* expression in lung adenocarcinoma normalized against the normal lung tissue of the same patient.

The analysis of the Oncomine Landi database (186) suggested no differences in *NOX1–3* and *NOX5* expression levels between normal and adenocarcinoma lung tissues (Fig. 13A), whereas *NOX4* expression was significantly increased in tumors. *SOD1* had markedly higher mRNA levels compared with *SOD2* or *SOD3*, the latter being significantly downregulated in adenocarcinoma tissues. Unlike that in several other tumors, the expression levels demonstrated relatively minor variation between individual patients. The percentage change in the expression levels compared to the normal lung supports the observation of minor patient-specific differences, excluding the expression of *NOX5* (Fig. 13B). Although the total expression levels of *NOX5* are low in individual patients (Fig. 13A), the increases or decreases vary from −192% to 426% (Fig. 13B), thereby suggesting that mutations and alterations in signal transduction pathways in lung adenocarcinoma markedly affect *NOX5* regulation.

## XVIII. ROS in Hematological Cancers

Hematological cancer cells are not in constant bidirectional interaction with tumor stroma, therefore representing an alternative model system for ROS studies. Self-renewal capacity, differentiation, aging, and the transformation of the most primitive HSCs are linked to ROS-derived DNA damage suggesting the multifaceted effect of redox enzymes in HSC fate. Interestingly, HSCs have been demonstrated to express several free radical-producing genes (286), although the HSC niche is characterized by hypoxia, which is one of the cornerstones in maintaining the primitive progenitor cell phenotype (56, 205). Importantly, ROS have been suggested to be a mediator in cell fate decision, in the induction of primitive hematopoietic progenitor cell differentiation and in the development of hematological malignancies (132, 149, 225, 226). In solid tumor development, ROS play a marked role in the induction of primary cell immortalization and transformation (39, 144) through the activation of oncogene-induced senescence and in the immortalization/transformation of primary cells. Interestingly, solid tumors have a mosaic senescent phenotype, suggesting the presence of cells that have lost the growth arrest signaling checkpoints—for example, through the downregulation of p21/p19/p16 signaling pathways (315). Correspondingly, in hematologic malignancies, the accumulation of ROS-derived DNA damage with consequent genomic aberrations has been suggested to demarcate the initiation and progression of the disease (310), although the function of ROS in hematological neoplasias is not completely characterized.

### A. ROS in CD34 HSC differentiation

HSCs, which originate from the mesoderm during ontogeny, differentiate into myeloid blood cell lineages (monocytes/macrophages, erythrocytes, neutrophils, basophils, eosinophils, platelets, and megakaryocytes) and lymphoid cell lineages (T cells, B cells, NK cells) (265). In mouse models, primitive hematopoietic progenitor cells have been classified into long-term hematopoietic stem cells (LT-HSCs), short-term HSCs, multipotent progenitor cells, and unipotent progenitor cells reflecting their self-renewal ability, cell cycling frequency, and differentiation status (248, 357).

**FIG. 14.**
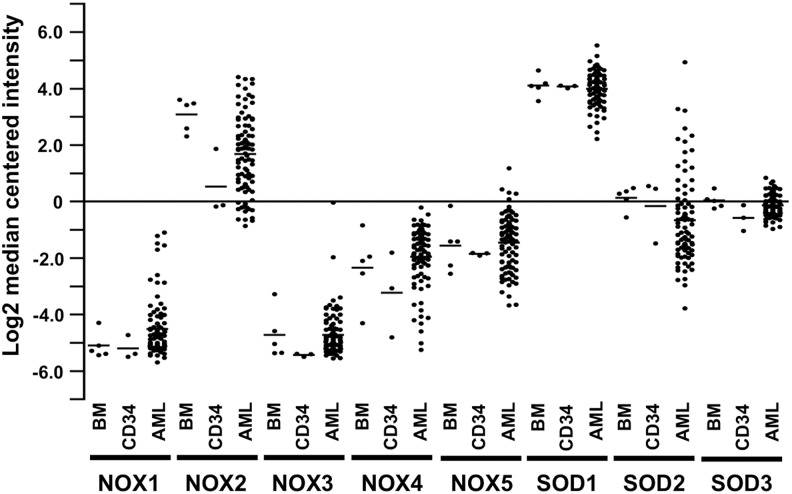
***NOX1–5*** **and** ***SOD1–3*** **gene expression in CD34^+^ cells and in AML extracted from Oncomine Valk database.** Gene expression values were collected from BM, peripheral blood CD34^+^, and AML cells. *NOX2*, *NOX4*, *NOX5*, *SOD2*, and *SOD3* redox gene expression values show differences between hypoxic BM and circulating CD34^+^ primitive hematopoietic progenitor cells. *NOX2* and *SOD1* show the highest expression values in collected samples. AML, acute myeloid leukemia; BM, bone marrow.

Although an emerging number of studies have suggested that ROS and ROS-related factors influence the development, self-renewal, migration, and differentiation of HSCs (145, 148, 149, 153, 286), HSCs have been demonstrated to express various redox enzymes (284, 286, 353) at relatively low levels. Oncomine Valk DNA microarray profiling of CD34^+^ bone marrow and peripheral blood HSCs from patients showed lower *NOX1*, *NOX3*, and *NOX4* mRNA expression than *NOX2*, *NOX*5, *SOD1*, *SOD2*, and *SOD3* expression, suggesting efficient O_2_^•−^ removal from HSCs (Fig. 14) (362). The hypoxic niche has been shown to coordinate low redox enzyme and ROS levels through hypoxia-inducible factor 1α (HIF-1α), a well-recognized redox gene expression regulator (169). Stabilization of cytoplasmic HIF-1α allows nuclear localization and consequent binding of HIF-1α to hypoxia response element (HRE) frequently found in the promoter region of redox genes (285). Physical tissue structures, such as arterial endothelial integrity, have been suggested to regulate the hypoxic environment by allowing or blocking extra- and intravasation of blood cells and serum components. Niches close to permeable sinusoidal arteries contain higher ROS concentrations, which correlate with a higher number of cycling and apoptotic primitive hematopoietic progenitor cells than niches located at less permeable arteries harboring the most primitive LT-HSCs (147).

Although the expression of ROS-producing enzymes is relatively low in the bone marrow HSC niche, it has been suggested that persistent genotoxic stress could lead to DNA damage accumulation in quiescent cells, eventually inducing their differentiation, aging, apoptosis, and malignant transformation (99, 373). The hypothesis was corroborated by recent studies, which demonstrated a correlation between increased ROS production, replicative stress, DNA damage, and premature senescence of stem cells in *in vivo* models. A high ROS content in grafted HSCs correlates with reduced repopulation ability in tertiary recipient mice, whereas HSCs with low ROS levels had significantly higher homing and engraftment capacity (132, 386). This was further supported by reports demonstrating that NAC treatment reduced the bone marrow ROS content and increased the human HSC transplantation efficiency in NOD-SCID mice (141, 146). Therefore, accumulated DNA damage could function as a physiological indicator of aging, affecting grafted stem cell homing and engraftment (291, 386).

Mechanistically, the accumulation of genomic damage is a result of the coordinated action of oxidative injuries, mutations caused by irradiation or chemicals, and aberrant DNA replication. In general, DNA damage activates the DNA damage response (DDR) signaling pathway, which is linked to increased ϒH2AX-ATM-CHK2-p53 DDR signal activation in the grafted cells, leading to the induction of cell cycle arrest with consequent DNA repair, differentiation, apoptosis, senescence, or transformation of HSCs (132, 303, 398). Interestingly, DNA damage may accumulate in the G0 phase into LT-HSCs due to low cell cycle frequency and consequent minimal DDR mechanisms, which are dependent on cell cycle-associated DNA replication. In the DNA injury correction process, the phosphorylation of p53 halts at cell cycling checkpoints G1/S and G2/M until DNA repair enzymes have corrected the mutations (54, 356). The hypothesis is in line with observations demonstrating markedly increased H2AX phosphorylation in HSCs isolated from old study subjects (303, 398).

Sources of ROS-induced DNA damage and HSC differentiation are incompletely characterized. The contribution of O_2_^•−^ and H_2_O_2_ has been linked to the malignant transformation of hematopoietic cells (135, 219, 296), although H_2_O_2_-producing SOD3 stimulates hematopoiesis by increasing primitive progenitor cell differentiation (1, 97). The contribution of SOD3 to HSC differentiation was causally supported by a study demonstrating that FOXO-null mice had reduced long-term repopulation ability. In these mice, *sod3* was identified as one of highly expressed leading edge genes (356), supporting the hypothesis that the enzyme can initiate the differentiation of HSCs by inducing DNA damage, maybe followed by activation of the differentiation process or, alternatively, stimulation of cell cycling and differentiation through the mitogenic RAS-ERK1/2 signal transduction pathway.

### B. ROS in hematological cancers and therapy

ROS-derived DNA damage is involved in the gradual progression of carcinogenesis, initiating from evolvement and amplification of self-renewing preleukemic HSC clones and leading to the development of AML or relapse after therapy (151). Mutations in plasma membrane-bound FLT3-ITD, a major genetic lesion leading to AML development, have been demonstrated to cause altered signal transduction, phosphorylation of STAT5, and consequent activation of RAC1, increasing ROS production. ROS-derived inactivation or decreased expression of phosphotyrosine phosphatases, such as SHP-1 and DEP-1/PTRRJ, may then contribute to transformation of 32D mouse hematopoietic progenitor cells and TF-1 human erythroblast (49, 111, 245).

STAT5 may also augment metabolic activity of myeloproliferative neoplastic cells by increasing the expression of 6-phosphofructo-2-kinase/fructose-2,6-biphosphatase 3 (PFKFB3) and by regulating JAK2V617F-dependent lactate production, metabolic activity, and glucose uptake (298). In chronic myelogenous leukemia (CML) cells, PI3K-AKT-GSK3β-β-CATENIN-MCL1 signaling has been linked to increased survival that directly correlates with increased ROS, especially H_2_O_2_, production. CML is frequently caused by translocation mutation between chromosomes 9 and 22 resulting in the formation of chimeric BCR-ABL kinase, which then stimulates synthesis of NADPH oxidase NOX4. Interestingly, NADPH oxidase NOX4-synthesized ROS inactivate phosphotyrosine phosphatase PP1α, thereby activating PI3K-AKT signaling and further strengthening CML cell drug resistance and cancer cell survival (252).

Oxygen radicals, DNA base modifications, and aberrant SOD and CATALASE expression contribute to the progression of chronic lymphocytic leukemia (CLL), acute lymphoplastic leukemia, monoclonal B lymphocytosis (MBL), a disease marked by B-cell expansion, and the CLL phenotype, which characteristically show increased levels of 8-oxo-2′-deoxyguanosine (8-oxo-dG) in lymphocytes and in urine. In a recent study, it was shown that the 8-oxo-dG mutation level in 29 MBL patients between 70 and 77 years and in 55 CLL patients between 63 and 77 years was significantly increased compared with that in 31 control patients (18, 60, 259, 264, 319, 400), therefore supporting the observations suggesting the early contribution of oxidative stress in hematological malignancies.

Further evidence supporting ROS-induced genetic aberrations was demonstrated in a study showing increased frequency of DNA double-strand breaks and nonhomologous genomic rearrangements in a myeloplastic syndrome (MS) mouse model. The authors suggested that ROS-derived genomic instability contributes to the clonal expansion of bone marrow CD34^+^ cells in *RAS*-*BCL2* mutation-harboring mice modeling MS/AML development. The involvement of ROS was confirmed by NAC treatment, which reversed DNA damage and error-prone DNA damage repair (296, 311). NAC primarily neutralizes the O_2_^•−^ radical, although it can also react with H_2_O_2_, therefore stressing the role of ROS in carcinogenesis.

However, the data extracted from the Oncomine Valk leukemia database consisting of gene expression data analyzed from 293 patients with AML highlight the expression of H_2_O_2_-producing SOD enzymes (Fig. 14) (362). The data demonstrate stable and low mRNA expression of *NOX1*, *NOX3*, *NOX4*, and *NOX5*, whereas the expression of *NOX2* is markedly higher and variable in different forms of cancer. Interestingly, the expression of *SOD* isoforms, especially *SOD1*, is clearly higher than that of *NOX* genes in all patient groups, suggesting SOD enzymes as a primary source of ROS in hematological malignancies. In general, the expression of *NOX* genes is increased in malignancies, whereas the expression of *SOD1*, *SOD2*, and *SOD3* is more variable, correlating with the differentiation status of the cells.

Irradiation can have a drastic impact on bone marrow stem cell clonal activity, affecting survival, expansion, mobilization, the ability to reconstitute the bone marrow niches, and differentiation of HSC clones (53, 193, 233, 255). In general, moderate or high irradiation can affect the primitive phenotype of stem cells by inducing differentiation, senescence, and apoptosis, or affecting the integrity of the stem cell niche (17, 77, 235) by increasing ROS production accompanied by oxidative DNA damage, with activation of ataxia-telangiectasia mutated (ATM)-CHK-p53-p21 and p38 MAPK cascades, the main signaling pathways induced by irradiation (282, 324).

The damage to the HSC niche occurs in two different modalities that are mediated by damaged cobblestone area forming colonies and by the disruption of the normal niche phenotype regulating stem cell proliferation and differentiation (179). Therefore, irradiation-derived ROS may have a marked influence on the HSC status on quiescent HSCs, modifying their stemness and even reflecting the aging of HSCs and might play a role in recovery of irradiation therapy patients (193, 208, 301, 387). Although the focus of ROS studies until now has been on DNA damage and the consequences of DNA injury on differentiation, aging, and on transformation of HSCs, ROS can activate *per se* cell signal transduction pathways, affecting the growth and survival of cells in an autocrine or a paracrine manner. As the HSC microenvironment contains redox enzyme expressing MSCs and/or MSC derivatives (208, 234, 241, 306), the role of the paracrine activity of niche cells could clarify further the role of ROS in HSC self-renewal and differentiation.

## XIX. Summary and Conclusions

Determining a universal measurable redox balance in cells is virtually impossible due to a high number of factors influencing the cellular redox state. Involvement of interacting cell signal transduction pathways may nevertheless assist cells to adapt to oxidative stress by coordinating oxidant and antioxidant gene expression, although they may also cause a differential response in cells, representing different diseases or differentiation degrees depending on the activation status of the signaling pathways. ROS production is most noticeably affected by GPCR activation, Ca^2+^ signaling, and H_2_O_2_-directed modification of the activation level of small GTPase RAC, which is an important subunit in NADPH oxidase NOX1, NOX2, and possibly also in NOX3. ROS-activated PI3K-AKT signaling is another compellingly interesting cascade because it is needed for p47^phox^ phosphorylation, which initiates the NAPDH oxidase complex assembly and might represent a key pathway in balancing H_2_O_2_ and O_2_^•−^ concentrations in cells.

The tissue environment provides additional uncharacterized factors that influence the overall redox gene expression, resulting in markedly high variation among patients. Therefore, the conclusions based on the data obtained from a large patient population may not be directly comparable with the results obtained from a single cell culture. Importantly, *in vitro* cell models may have limitations in modeling cancer depending on the mutations in oncogenes, activation status of the signaling pathways, and the differences in mechanisms of individual redox gene induction, which then influence redox gene expression. Although the data observed in the cell culture model systems have unquestionably demonstrated the role of O_2_^•−^ and H_2_O_2_ in tumorigenesis, the differences in the *in vitro* and *in vivo* model systems used could explain some contradictory conclusions in the function of redox enzymes in cancer.

When evaluating the data observed in the model systems, it is crucial to distinguish the differential signaling effects of ROS, especially those of H_2_O_2_, which directly correlate to the level of ROS. This, in turn, affects the use of redox genes for diagnostic purposes. Although some redox genes could be considered supplemental candidate markers in carcinogenesis, the individual discrepancy in the mRNA expression levels in patients invalidates their reliability in prognostic diagnosis. However, the systemic identification of signaling pathways affecting simultaneously the activation of redox genes or mediating the ROS effect may offer potential novel drug targets in cancer treatment or complement existing clinical protocols.

## Abbreviations Used

8-oxo-dG: 8-oxo-2′-deoxyguanosine
AA: arachidonic acid
ABL: Abelson murine leukemia viral *oncogene* homologue
ALK: anaplastic lymphoma kinase
ALS: amyotrophic lateral sclerosis
AML: acute myeloid leukemia
AMPK: 5′ adenosine monophosphate-activated protein kinase
APC: adenomatous polyposis coli
AQP3: aquaporin 3
ATM: ataxia-telangiectasia mutated
AXIN: axis inhibition protein 2
BCAR1: breast cancer anti-estrogen resistance protein 1
BIM: Bcl-2 interacting mediator of cell death
BRCA1/2: breast can½ 1/2
Ca^2+^: calcium
CAMKII: calcium/calmodulin-dependent kinase II
CK1: casein kinase
CLL: chronic lymphocytic leukemia
CML: chronic myelogenous leukemia
COX2: cyclooxygenase 2
CREB: cAMP response element-binding protein
CXCR4: C-X-C chemokine receptor type 4
cyt b588: cytochrome b588
DAG: diacylglycerol
DDR: DNA damage response
ECM: extracellular matrix
EGF: epidermal growth factor
EMT: epithelial/mesenchymal transition
FAD: flavin adenine dinucleotide
FAK: focal adhesion kinase
FAP: familial adenomatous polyposis
FLT3-ITD: FMS-like tyrosine kinase 3 with internal tandem duplications
FOX: forkhead box
GAP: small GTPase activator protein
GDI: guanine nucleotide disassociation inhibitor
GEF: guanine nucleotide exchange factor
GPCR: G protein-coupled receptor
GSK3β: glycogen synthase kinase 3β
H_2_O_2_: hydrogen peroxide
HER2: human epidermal growth factor receptor 2
HIF-1α: hypoxia inducible factor 1α
HNPCC: hereditary nonpolyposis colorectal cancer
HSC: hematopoietic stem cell
IκBα: nuclear factor of kappa light polypeptide gene enhancer in B cell inhibitor α
ICAM: intercellular adhesion molecule
IFN-γ: interferon gamma
IGF: insulin growth factor
IKKα: inhibitor of nuclear factor kappa-B kinase subunit α
IL: interleukin
IP3: inositol triphosphate
iPSC: induced pluripotent stem cell
IRAK: interleukin-1 receptor-associated kinase
JNK: c-Jun N-terminal kinase
LIMK: LIM kinase
LMW: low-molecular-weight
LOX: lipoxygenase
LT-HSC: long-term hematopoietic stem cell
MBL: monoclonal B lymphocytosis
MCL1: myeloid cell leukemia 1
MMP: matrix metalloproteinase
mRNA: messenger RNA
MS: myeloplastic syndrome
MSCs: mesenchymal stem/stromal cells
NAC: N-acetyl cysteine
NFκB: nuclear factor kappa-light-chain-enhancer of activated B cells
NK: natural killer
NOXA1: NADPH oxidase activator 1 subunit
NOXO1: NADPH oxidase organizer 1 subunit
NSCLC: nonsmall-cell lung cancer
O_2_^•−^: superoxide anion
PAF: platelet-activating factor
PDGF: platelet-derived growth factor
PD-L: programmed death ligand
PDPK1: 3-phosphoinositide-dependent protein kinase-1
phox: phagocytic oxidase
PI3K: phosphatidylinositol-4,5-bisphosphate 3-kinase
*PI3Kα*: phosphatidylinositol-4,5-bisphosphate 3-kinase catalytic subunit α
PIP2: phosphatidylinositol (4,5)-biphosphate
PIP3: phosphatidylinositol (3,4,5)-triphosphate
PKA: protein kinase A
PKC: protein kinase C
PLC: phospholipase C
PMA: phorbol 12-myristate 13-acetate
PTC: papillary thyroid cancer
PtdIns: phosphatidylinositol
PTEN: phosphatase and tensin homologue
PTK2β: protein tyrosine kinase 2β
PTP: protein tyrosine phosphatase
PTPRJ/DEP-1: protein tyrosine phosphatase, receptor type J
redox: reduction/oxidation
RGS4: regulator of G protein signaling 4
RNAi: RNA interference
ROCK: RHO-associated, coiled-coil-containing protein kinase
ROS: reactive oxygen species
RTK: tyrosine kinase receptor
SCLC: small-cell lung cancer
SD: standard deviation
SDF-1: stromal-derived factor 1
SH: SRC homology
SOD: superoxide dismutase
SP1: specificity protein 1
STAT5: signal transducer and activator of transcription 5
TGFβ: transforming growth factor β
TKS4: tyrosine kinase substrate with 4 SH3 domains
TKS5: tyrosine kinase substrate with 5 SH3 domains
TLR4: Toll-like receptor 4
TNFα: tumor necrosis factor alpha
TRAF6: TNF receptor-associated factor 6
TRAIL: TNF-related apoptosis-inducing ligand
TSH: thyroid-stimulating hormone
TSHR: thyroid-stimulating hormone receptor
UTR: untranslated region
UV: ultraviolet
VEGF: vascular endothelial growth factor
WNT: wingless-type MMTV integration site family
WWTR1: WW domain containing transcription regulator 1
ZEB2: zinc finger E-box binding homeobox 2
